# Climate change leads to significant loss of soil inorganic carbon

**DOI:** 10.1093/nsr/nwag075

**Published:** 2026-02-06

**Authors:** Jin Hu, Zelin Huang, Zhongxiu Sun, Xiaodong Song, Yuanyuan Huang, Kazem Zamanian, Feng Tao, Fei Yang, Huiying Wen, Ganlin Zhang

**Affiliations:** State Key Laboratory of Lake and Watershed Science for Water Security, Nanjing Institute of Geography and Limnology, Chinese Academy of Sciences, Nanjing 211135, China; College of Advanced Agricultural Sciences, University of Chinese Academy of Sciences, Beijing 100049, China; State Key Laboratory of Soil and Sustainable Agriculture, Institute of Soil Science, Chinese Academy of Sciences, Nanjing 211135, China; College of Land and Environment, Shenyang Agricultural University, Shenyang 110866, China; College of Advanced Agricultural Sciences, University of Chinese Academy of Sciences, Beijing 100049, China; State Key Laboratory of Soil and Sustainable Agriculture, Institute of Soil Science, Chinese Academy of Sciences, Nanjing 211135, China; Key Laboratory of Ecosystem Network Observation and Modeling, Institute of Geographic Sciences and Natural Resources Research, Chinese Academy of Sciences, Beijing 100101, China; State Key Laboratory of Resources and Environmental Information System, Institute of Geographic Sciences and Natural Resources Research, Chinese Academy of Sciences, Beijing 100101, China; Institute of Earth System Sciences, Section Soil Science, Leibniz University of Hanover, Hanover 30419, Germany; Department of Ecology and Evolutionary Biology, Cornell University, Ithaca, NY 14853, USA; State Key Laboratory of Soil and Sustainable Agriculture, Institute of Soil Science, Chinese Academy of Sciences, Nanjing 211135, China; College of Advanced Agricultural Sciences, University of Chinese Academy of Sciences, Beijing 100049, China; State Key Laboratory of Soil and Sustainable Agriculture, Institute of Soil Science, Chinese Academy of Sciences, Nanjing 211135, China; State Key Laboratory of Lake and Watershed Science for Water Security, Nanjing Institute of Geography and Limnology, Chinese Academy of Sciences, Nanjing 211135, China; College of Advanced Agricultural Sciences, University of Chinese Academy of Sciences, Beijing 100049, China; State Key Laboratory of Soil and Sustainable Agriculture, Institute of Soil Science, Chinese Academy of Sciences, Nanjing 211135, China

**Keywords:** soil inorganic carbon, climate change, China, process-based model, carbonate

## Abstract

Soil inorganic carbon (SIC) pools are comparable in size to soil organic carbon pools and are vulnerable to climate change; however, SIC responses to climate change remain uncertain because of the lack of process-based simulations. Here, we developed a new process-based model integrating daily water balance dynamics with carbonate chemical equilibria at a 10 cm vertical resolution to predict the effects of climate change on the SIC pool down to a soil depth of 2 m in China until 2100. We found that across the four shared socioeconomic pathways, SIC stock in China’s topsoil (0–10 cm) decreased by 314 ± 8 Tg C, accompanied by a loss of 217 ± 9 Tg C from the 2 m soils. These findings challenge the traditional view of SIC stability in terrestrial carbon cycles, reveal substantial losses of SIC in both topsoils and deep soils, and highlight the projection of future climate and global inorganic carbon cycle feedback.

## Teaser

A new process-based model projects that climate change may lead to an ∼8% reduction in soil inorganic carbon (SIC) from China’s topsoil (0–10 cm) by 2100, challenging the paradigm of SIC as a stable carbon pool.

## INTRODUCTION

Soil inorganic carbon (SIC)—primarily in the form of carbonate minerals such as calcium carbonate (CaCO_3_)—is the dominant carbon (C) pool in more than half of the world’s soils, storing an estimated 2305 petagrams (Pg) of C within the top 2 m [1,2]. For decades, this vast reservoir has been regarded as geochemically stable, with residence times on the millennia scale under natural conditions. Consequently, SIC has often been overlooked in terrestrial C cycle frameworks [3]. However, emerging evidence now challenges this long-standing paradigm, revealing the unprecedented sensitivity of SIC pools to contemporary environmental perturbations [1,4,5]. Recent studies have projected potential loss exceeding 23 Pg SIC over the next 30 years [1]. Climate-induced alterations in hydrological regimes and carbonate mineral reactivity drive water movement, thereby playing a pivotal role in the turnover of SIC [6–9]. Given the substantial role of SIC dynamics in the long-term carbon cycle and its dual role as both a carbon sink and a climate feedback agent, it is urgent to simulate regional SIC transformation and persistence under changing climatic conditions based on physicochemical pathways and regulatory mechanisms.

A mechanistic understanding of SIC turnover is essential for refining global C budgets and developing robust climate mitigation strategies [10]. Although interest in the spatiotemporal dynamics of SIC has grown [1–3,9,11,12], current research remains dominated by laboratory experiments, statistical models and data-driven analyses, which may overlook the coupled climatic and geochemical feedbacks controlling SIC turnover [13]. Assessments were generally conducted under climatic conditions of the late 20th century and investigated the processes of CaCO_3_ dissolution and precipitation (Table S1), limiting their applicability to current and future scenarios [5]. Recent investigations into SIC accumulation have utilized compartmentalized 1D models by incorporating hydrological processes; however, these

models are limited by few soil profile data (*n* = 16) and simplified carbonate equilibrium that neglect critical acid‒base interactions and saturation dynamics that are essential for carbonate equilibrium [6]. The dissolution of CaCO_3_ under natural conditions involves the H_2_O–CO_2_ system (Methods, Equations 1 and 2) and can be quantified by well-established geochemical reactions [6,14–17]. The geochemical equilibrium framework of the CaCO_3_–CO_2_–H_2_O system can provide critical insights of carbonate dynamics modeling [15,18]. However, this framework has rarely been applied to soil systems and remains absent from regional assessments and long-term simulations. The central challenge of SIC turnover modeling is the inadequate integration of carbonate geochemical equilibria with hydrologically driven translocation along soil profiles, which motivated the development of a new framework to address this fundamental gap (Supplementary data, Text S1). This integration is essential for capturing the spatiotemporal variations in SIC at the regional-scale dynamics and can advance the mechanistic understanding of soil carbon sequestration under climate change.

**Figure 1.**
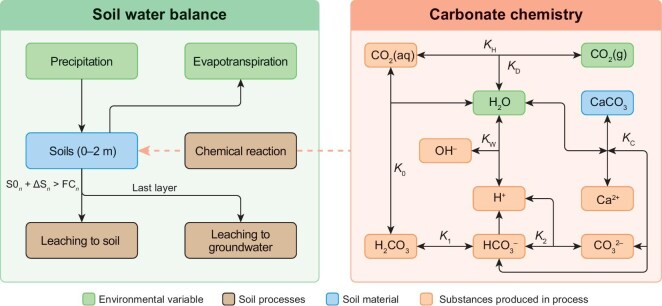
The soil inorganic carbon turnover model (SINOCOM). S0 denotes the initial soil moisture content in each soil layer. ΔS represents the soil moisture surplus, defined as the net balance of precipitation minus evapotranspiration. *n* indicates the number of discrete soil layers in a vertical soil profile. FC represents field capacity. The orange dashed arrow illustrates couplings between the soil-water-balance module and the carbonate-chemistry module. Letters labeled along reaction arrows in the carbonate-chemistry module denote equilibrium constants governing carbonate dissolution and precipitation reactions, with parameter values detailed in Table S2. *K*_0_ is the equilibrium constant for the H_2_CO_3_ and CO_2_. *K*_1_, *K*_2_ and *K*_W_ are the dissociation constants of H_2_CO_3_, HCO_3_^−^ and H_2_O, respectively. *K*_C_ indicates the solubility product of CaCO_3_. *K*_D_ is the partition coefficient of CO_2_ between the gaseous and aqueous phases. *K*_H_ denotes Henry’s law constant.

Here, we develop a novel process-based Soil Inorganic Carbon Turnover Model (SINOCOM) to quantify the effects of climate change on SIC dynamics from 2015 to 2100 across China (Methods, Fig. 1). In this study, the loss of SIC refers to the movement from specific soil layers [7,13,19–21]. The model integrates a physically based soil-water-balance module with carbonate geochemical equilibrium, excluding acidification processes, to isolate climate-driven effects on SIC. The water-balance module regulates SIC leaching and accumulation through precipitation and evapotranspiration. The carbonate-equilibrium module exerts its influence through temperature, net primary productivity (NPP) and CO_2_-driven carbonate dissolution and precipitation reactions (Fig. 1). To the best of our knowledge, SINOCOM might be the first process-based SIC model that comprehensively integrates climate change, hydrological processes and carbonate chemical-equilibrium modules. Our study simulates the daily SIC dynamics under four shared socioeconomic pathways (SSPs) across 10 cm depth intervals within 2 m soils, incorporates key environmental drivers, and constrains model uncertainty through parameter range. These advances clarify the climatic controls on SIC redistribution, quantify the relative contributions of SIC vertical translocation and hydrological export, and may provide a mechanistic foundation for improving predictions of SIC dynamics under climate change.

## RESULTS AND DISCUSSION

### Overall changes in SIC

**Figure 2.**
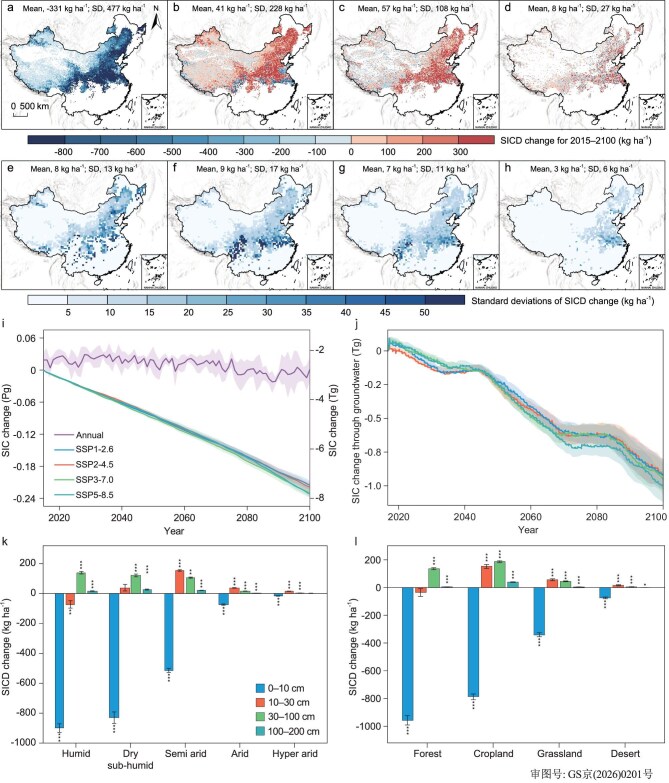
Dynamics of SIC under climate change from 2015 to 2100. (a–d) Spatial distribution of changes in SICD at 0–10 cm (a), 10–30 cm (b), 30–100 cm (c) and 100–200 cm (d). (e–h) Standard deviations of changes in the SICD based on 100 simulations corresponding to the same groupings as in (a–d). (i) Daily and annual variations in SIC within the 0–100 cm soil layer. (j) Daily dynamics of SIC loss through groundwater within the 0–100 cm soil profile. Shaded areas represent ±1 SD. (k and l) Total change in SICD under different climatic conditions (k) and land use (l). Climatic condition classifications are provided in Fig. S5. The data of (a–h, k and l) are simulations based on SSP2-4.5. In (k and l), independent sample *t*-tests are conducted, in which *, ** and *** indicate significant difference with *P* < 0.05, *P* < 0.01 and *P* < 0.001, respectively. The error bars indicate the standard errors.

A substantial decline in total SIC of 209–225 Tg C in 2 m soils in China was projected from 2015 to 2100 (Fig. 2a–d and Fig. S1) under four SSPs (Methods, Text S2 and S3). The total SIC loss of 307–321 Tg C occurred in the topsoil (0–10 cm), in which the changing rates declined with soil depth substantially (Fig. S2, Table S3). The formation of new pedogenic carbonates (∼4% relative to total SIC loss) was found; however, despite this formation, the net loss of SIC remained under all the climatic conditions (Fig. S3, Table S4). The SIC of topsoil will be depleted in ∼9% of regions (Fig. S4), predominantly in areas with high soil moisture availability, accounting for ∼25% of fertilizer-induced SIC loss in croplands [22]. The depletion of SIC in topsoil diminishes soil acid buffering capacity, thereby undermining the resilience of soil functions essential for ecosystem health and global carbon cycle stability [22–24].

We coupled the process-based SINOCOM model, at a spatial resolution of 0.5° × 0.5° and a daily temporal resolution with machine-learning downscaling (Methods), thereby transforming simulations into 1-km SIC maps (Fig. 2a–d, Fig. S1), with a predictive performance with R^2^ values of 0.85 to 0.91 (Tables S5 and S6). In addition, the model was run 100 times with changing parameters, in which the standard deviation was calculated and its spatial distribution was adopted to assess model uncertainty (Fig. 2e–h, Methods). Uncertainties in total SIC losses were estimated at 8 Tg C for topsoils (0–10 cm) and 9 Tg C for soils to 2 m depth, corresponding to approximately 2%–4% of the projected SIC losses. High uncertainty was found in some humid areas (Fig. 2a–h, Fig. S5), illustrating that strong hydro gradients, intensive carbonate dissolution and complex interactions between different leaching intensities reduce model stability. We further partitioned the model uncertainty between the water-balance and carbonate-chemical-equilibrium modules (Fig. S6). Water-balance uncertainty dominated SIC turnover variability in the top 0–30 cm (∼60%) compared with deeper layers (∼38%), whereas uncertainty from carbonate equilibrium remained relatively stable across the profile (∼52%). A comparison with a previously published observational dataset revealed that the model captured spatial variation in SIC, with R^2^ values ranging from 0.35 to 0.45 (Fig. S7). The change in the SIC density (SICD) was consistent with regional-scale assessments of SIC loss [1,2,22], and together these findings support the reliability of the SINOCOM simulations.

**Figure 3.**
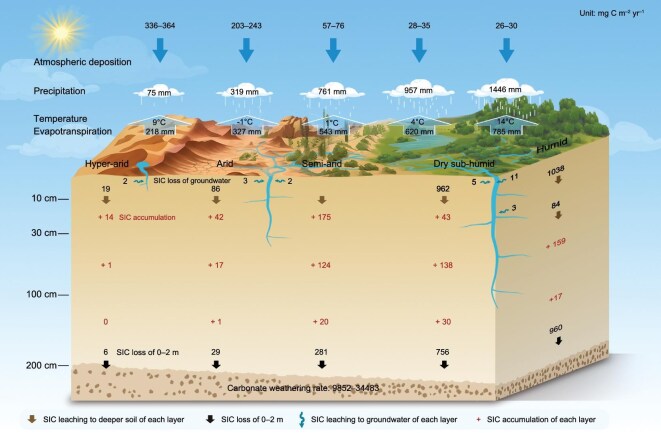
Projections of depth-specific SIC change rates under different climatic conditions by the end of the 21st century. SIC change rates of soil depths are shown, attributed to vertical leaching and lateral export via groundwater. The carbonate rock-weathering rate was calculated by Zeng *et al*. [32] and Goldscheider *et al.* [33]. Values represent SIC translocation fluxes (mg C m^−2^ year^−1^), except the precipitation, evapotranspiration and temperature. Black and red numbers indicate SIC loss and SIC accumulation in specific layers, respectively. Annual climate data (i.e. precipitation, temperature and evapotranspiration) and atmospheric deposition rate from 2015 to 2100 are from the CMIP6 dataset and Jeong [34].

SIC exhibited a decreasing trend across all the climate scenarios (Fig. 2i and j), with minor interannual variability induced by climate fluctuations. Total topsoil SIC loss was greatest under SSP5-8.5 (321 Tg C, 8.5%) (*P* < 0.05), followed by SSP3-7.0 (317 Tg C, 8.3%), SSP2-4.5 (312 Tg C, 8.2%) and SSP1-2.6 (307 Tg C, 8.1%) (Table S3). These patterns can be attributed to the small changes in mean annual precipitation, which differed by only approximately 30 mm between the wettest and driest scenarios (Fig. 3, Fig. S8), in combination with the inherently slow turnover rate of SIC (Methods, Equations 1 and 2).

### Pathways of SIC loss

Approximately 1% of topsoil SIC was lost through groundwater into aquatic systems (lateral export), whereas 29%–31% of SIC was leached and accumulated in 10–200 cm soil layers, and the remaining 68%–70% was leached out of the 200 cm soil layer (vertical translocation) (Table S3). SSP5-8.5 resulted in greater SIC loss via lateral flow than those in other scenarios (Fig. 2i and j), which are characterized by severe soil erosion due to increased precipitation frequency or intensity [25]. Groundwater-mediated SIC loss depended on soil depth, because of the variability in soil thickness, with shallow soils under arid conditions having limited lateral water flow compared with deeper soils under humid conditions [26,27]. Under arid and semi-arid conditions, SIC transported by groundwater flow was more likely to accumulate in closed inland water bodies and lakes, where it can re-precipitate and continue to cycle within the regional carbon pool [28,29]. In contrast, in humid conditions, groundwater-mediated SIC fluxes were more likely to be exported from the region and contributed to the long-term geochemical carbon cycle as part of large-scale transport processes [30]. SIC loss driven by downward leaching and potential re-precipitation in humid areas was more than that in hyper-arid conditions within the 2 m soils (Figs 2 and 3). These findings highlight climate as an important factor regulating SIC translocation and redistribution within the soil profile [7,9,31].

### Effect of climate and land use on SIC loss

Semi-arid regions experienced more severe total topsoil SIC loss (124 Tg C, 10.5%) than those in humid (107 Tg C, 51.7%), dry sub-humid (63 Tg C, 40.0%), arid (16 Tg C, 1.1%) and hyper-arid (1 Tg C, 0.2%) regions (Fig. 3, Fig. S9a, Table S7). It was suggested that the ecological environment may deteriorate in these semi-arid regions with substantial SIC losses, and region-specific carbon management strategies should be prioritized, to address the spatially heterogeneous responses of SIC to climate forcing, a prerequisite for effective mitigation and adaptation [8]. These areas, which are characterized by fragile soil structures and limited water availability, may face heightened risks of soil degradation, reduced fertility and desertification [9,35]. Efforts to increase soil carbon sequestration should consider the stabilization of inorganic carbon, particularly in vulnerable semi-arid and arid landscapes [12].

SIC loss also varied significantly across land use (Fig. S9b, *P* < 0.05). The greatest amount of total SIC loss was found in croplands (104 Tg C, 17.2%) from tillage-induced soil mixing, followed by grasslands (92 Tg C, 6.7%) linked to deep root-enhanced leaching, forests (77 Tg C, 38.6%) due to organic matter stabilization, and deserts (14 Tg C, 1.0%) with physically protected carbonates (Fig. 2l, Table S8). This variability follows a gradient that can be explained by the distinct climatic conditions, as well as land use [31]. These results are consistent with recent continental- and global-scale assessments reporting significant SIC loss under climate change scenarios, as well as loss induced by atmospheric nitrogen deposition and anthropogenic reactive nitrogen inputs, particularly in arid and semi-arid regions [2,36,37]. Previous studies estimate that approximately 19.12%–19.47% of China’s SIC stocks will be lost by 2100 as a result of nitrogen deposition and climate change [2]. In comparison, the SIC losses quantified in our study account for ∼13% of these projected declines. In addition, observational evidence suggests that enhanced nitrogen deposition, fertilizer-induced soil acidification and reduced calcium inputs associated with declining base-cation deposition have driven an SIC loss of approximately 0.03 Pg C year^−^^1^ across China [36], of which our estimated SIC losses comprise ∼10%.

### Precipitation and its seasonal variation regulate SIC leaching

**Figure 4.**
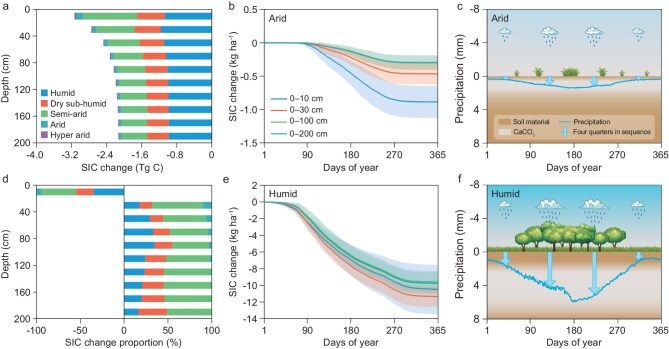
SIC leaching and accumulation patterns in soil profiles from 2015 to 2100. (a and d) Total cumulative (a) and absolute proportional (d) changes in SIC stocks at 20 cm depth intervals under different climate conditions. (b and e) Mean daily cumulative changes in SIC stocks under arid (b) and humid (e) climates. The slope of each curve represents the SIC loss rate, while the shaded areas denote the confidence intervals derived from the standard deviations over the period of 2015–2100. (c and f) Conceptual models of SIC leaching and accumulation patterns in soil profiles under arid (c) and humid (f) climates. To better illustrate SIC variations with depth, both panels (a and d) present data in 20 cm increments; the results at 10 cm intervals are shown in Fig. S10. The blue line represents the average daily precipitation. The blue arrows indicate the direction of water-driven transport of SIC within the soil profile in annual quarters. The gray zones within the soil profiles represent schematic redistribution patterns of SIC with depth.

The depth and spatial extent of SIC leaching exhibited strong climatic gradients (Fig. 4a and d; Figs S10 and S11). While SIC leaching reached 200 cm in humid to semi-arid regions, it was confined to 100–150 cm under arid and hyper-arid conditions (Table S9, Fig. S11). The pattern reveals a close relationship between precipitation and SIC redistribution depth, with SIC accumulating close to the land surface under dry conditions (Figs 3 and 4, Fig. S11).

To further examine the temporal dynamics of SIC turnover, the cumulative daily SIC losses were quantified from 2015 to 2100. Given that SIC losses under hyper-arid conditions were negligible, arid systems exhibited the lowest SIC loss with an approximately steady dynamic (Table S7). Humid and arid climates were selected to capture SIC responses to precipitation seasonality, with precipitation serving as the primary driver regulating the downward translocation of SIC [38]. We found that the temporal dissolution of SIC fluxes revealed stark seasonality. Precipitation peaked during the summer months (June–August, Fig. 4c and f). SIC loss remained low from December to February, increased markedly from March to August, and peaked in mid-summer, followed by a gradual decline (Fig. 4b and e). Warm-season precipitation (March to August) in arid climates accounted for 68% of the mean annual input (204 mm) and resulted in 85% of the mean annual SIC loss (Fig. 4b). Similarly, intense seasonal precipitation (798 mm, 71% of mean annual input) promoted 76%–81% of the mean annual SIC loss in humid conditions.

Our process-based simulation revealed that seasonal precipitation variability was the critical driver for SIC leaching. The depth and intensity of SIC accumulation were strongly influenced by the timing and magnitude of seasonal precipitation [13], with warm-season events mobilizing carbonates to deeper depths through episodic dissolution‒reprecipitation cycles [38–41]. This process of seasonal influence aligns with the evidence from pedogenic carbonate formation [39,40]. These findings challenged previous empirical models that emphasized mean annual precipitation as the sole predictor [3,7,13].

### Sensitivity analysis

**Figure 5.**
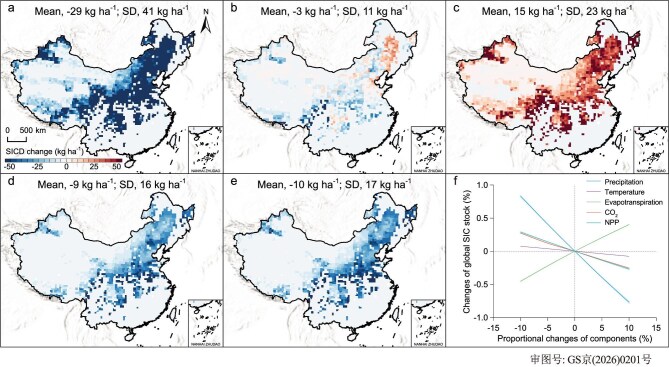
Sensitivity of the SIC to climate factors. (a–e) Spatial response patterns of SICD in the top 10 cm to a 10% increase in individual environmental variables, including precipitation (a), temperature (b), evapotranspiration (c), CO_2_ concentration (d) and NPP (e). (f) Relative variation in SIC stocks in relation to proportional scaling of key parameters, representing the responsiveness of SIC dynamics to sensitivity and uncertainties in parameterization. Shaded areas denote the standard deviation across simulations under the four climate scenarios.

Our simulations highlighted the sensitivity of SIC stocks to climate changes during the 21st century. Sensitivity analysis revealed that topsoil SIC was the most sensitive to climate change, with responses decreasing with depth (Fig. S12). Precipitation was the primary climatic driver of SIC turnover, with ±10% precipitation changes causing total topsoil SIC variations ranging from −7.7‰ to 8.3‰ (−27 Tg C to 29 Tg C). This effect was nearly twice that of evapotranspiration (−4.5‰ to 4.0‰, −16 Tg C to 14 Tg C), more than three times greater than that of the atmospheric CO_2_ concentration (−2.5‰ to 2.7‰, −9 Tg to 10 Tg C) and NPP (−2.7‰ to 2.9‰, −9 Tg C to 10 Tg C) and more than 10 times the influence of temperature (−0.8‰ to 0.7‰, −3 Tg C to 3 Tg C) (Fig. 5). The spatial sensitivity of the SIC in the topsoil to climatic drivers strongly responded to a 10% increase in individual environmental variables under semi-arid conditions (1–13 Tg C). However, humid (1–6 Tg C) and dry sub-humid (1–5 Tg C) conditions exhibited low sensitivity to climatic variables owing to the low content or absence of SIC, whereas hyper-arid (less than 1 Tg C) and arid (0–3 Tg C) conditions exhibited minimal responses because of limited water movement (Fig. 5a–e) [42].

The response of the SIC to precipitation and evapotranspiration reflects the direct control of water-balance processes in regulating SIC leaching and accumulation [38]. In contrast, carbonate-equilibrium processes, governed by the CO_2_ concentration, NPP and temperature, indirectly control SIC dissolution and precipitation [18,32]. In the water-balance module, precipitation directly enhanced vertical water movement, driving SIC leaching throughout the soil profile. Intensified precipitation regimes and increased hydrological connectivity promote carbonate leaching, facilitating the downward translocation of bicarbonate and divalent cations [38,43]. The spatial pattern of SIC sensitivity to precipitation closely matched that of total SIC loss (Fig. 5a and 2), underscoring the dominant role of precipitation in SIC turnover [42]. In contrast, evapotranspiration inversely modulated these effects by reducing soil moisture availability. SIC is stabilized through limited leaching and strong evaporative concentration. Thus, the SIC sensitivity pattern in response to evapotranspiration was the inverse of that to precipitation [13]. The soil CO_2_ concentration, NPP and temperature varied across different water availability levels (Fig. 5b, d and e). CO_2_ acted as both a reactant and a product in carbonate dissolution/precipitation reactions, and temperature influenced each step of the reaction sequence [9]. Elevated soil CO_2_ concentrations increase the production of bicarbonate, and CO_2_ solubility inversely varies with temperature (Table S2) [15], whereas soil CO_2_ partial pressure might directly increase because of increased microbial activity [44]. The SIC response to NPP exhibited a spatial pattern similar to that of the air CO_2_ concentration (Fig. 5d and e) because NPP regulates the amount of organic carbon fixed by vegetation and thereby alters soil carbon dioxide concentrations. Moreover, climate-induced vegetation shifts modify organic inputs, further enhancing carbonate mobilization. On the other hand, temperature has a dominant effect on SIC dissolution [15]. Increased temperatures can reduce the soil CO_2_ concentration to some extent, which may in turn lessen SIC loss. However, warming simultaneously disrupts pedogenic carbonate formation by weakening evapotranspiration-driven geochemical controls, thereby constraining long-term SIC stabilization [44]. Therefore, complex coupling between these variables controls the carbonate equilibrium system, which influences both the dissociation of carbonate species and CaCO_3_ precipitation [6,45].

### Limitations and future research

In this study, SINOCOM was shown to effectively simulate SIC dynamics by integrating daily water-balance dynamics with carbonate chemical equilibria at a 10 cm vertical resolution. However, a limitation of the current framework is that it accounts for only the influence of NPP on soil CO_2_. Roles of vegetation and soil microbes in shaping terrestrial carbon pools are found [44,46,47]. Vegetation cover and fungal biomass explain a proportion of SIC variability [44]. The SIC links long-term geological processes with short-term biogenic fluxes; capturing the biological regulation of soil CO_2_ is essential. Microbial respiration and root activity strongly influence soil CO_2_ concentrations, which affect carbonate solubility and the direction of inorganic carbon transformation [48]. Unfortunately, these processes cannot be incorporated into SINOCOM due to data limitations and the lack of a consensus on the mechanisms by which plants and microbes drive SIC dynamics. In the near future, long-term observation experiments should be conducted to collect modeling parameters of microbial and rhizosphere regulation of soil CO_2_ to quantify their overall effect on SIC turnover.

Future development may integrate SINOCOM within Earth system models to quantify the interactions between SIC and vegetation dynamics, acid input and mineral weathering [13]. The model could also explicitly consider acid inputs from both atmospheric deposition and anthropogenic sources, as well as the complex interactions within the CaCO_3_–CO_2_–H_2_O system in response to acid. Dry deposition of CaCO_3_, such as dust inputs, may be parameterized based on long-term observational data. Continuous silicate weathering, which regulates atmospheric CO_2_ over geological timescales, may likewise be incorporated [23,49]. An updated model could incorporate CO_2_ produced by root and microbial respiration and account for biotic modifications of calcium and magnesium availability, capturing SIC loss induced by acid inputs and SIC formation driven by calcium additions, enabling more accurate simulations of SIC variability. Such coupling allows explicit evaluation of SIC feedback on global carbon budgets and strengthens the representation of coevolving inorganic and organic carbon processes during soil development [32,50,51]. Although some parameters in driving SIC turnover have not been incorporated, SINOCOM integrates key soil hydrology and carbonate chemistry parameters to quantify SIC turnover processes, yielding credible insights into climatic controls. By resolving the spatiotemporal complexity of SIC dynamics, SINOCOM provides a basis for integrating inorganic carbon processes into Earth system models with the potential to refine global carbon storage estimates and inform climate mitigation strategies.

## METHODS

Detailed methods are given in the Supplementary data (Text S3).

## Supplementary Material

nwag075_Supplemental_File

## Data Availability

The data used to produce Figs 1–5 and Figs S1–S15 are available via Figshare at https://figshare.com/s/94d57494e4748107516e.
