# Supplementary material for: Climate change leads to significant loss of soil inorganic carbon
Source: Natl Sci Rev. 2026 Feb 6;13(5):nwag075. doi: 10.1093/nsr/nwag075 (PMC12993447; doi:10.1093/nsr/nwag075)
Supplement: nwag075_Supplemental_File [file nwag075_supplemental_file.pdf]

Supplementary Materials for
Climate change leads to significant loss of soil inorganic carbon

Supplementary Text 1: Comparative analysis of existing SIC models and advancements through SINOCOM

Over the past six decades, SIC models have progressively evolved, thereby incorporating increasingly sophisticated representations of carbonate turnover mechanisms, hydrological processes, and spatiotemporal dynamics (Table. S1). Early models, such as those developed by Arkley [1] and Rogers [2], focused primarily on the empirical relationships between soil carbonate stratification and climate variables. However, these models generally lacked explicit chemical formation mechanisms and did not resolve vertical or spatial variations, which limited their ability to capture subsurface carbonate processes and spatial heterogeneity. In addition, key hydrological drivers, which are now recognized as critical in driving soil carbon cycling, were typically omitted. Later models introduced more detailed processes, including dust-driven calcium inputs and stochastic rainfall variability [3–9]. However, most of these methods remained constrained by thermodynamic assumptions and often operated at coarse temporal or vertical resolutions. Importantly, the absence of coupled land hydrology in these models restricted their ability to fully represent terrestrial carbon dynamics. The development of the SLIC model represented a meaningful step forward by introducing daily time steps and stochastic rainfall patterns; however, it still did not account for detailed carbonate dissolution dynamics or groundwater carbon fluxes [10]. While these early models provided valuable conceptual foundations, they lacked the process-level integration and spatial detail needed for modern Earth system applications.

In this study, we present SINOCOM, a new SIC modeling method that substantially advances previous approaches. Unlike earlier models that employed simplified thermodynamic or kinetic schemes, SINOCOM integrates a detailed chemical system that simultaneously simulates carbonate precipitation and dissolution under thermodynamic equilibrium, dynamically resolves acid–base reactions to track pH variations throughout soil profiles, and explicitly represents the stepwise processes of carbonate dissolution and saturation [11]. Notably, SINOCOM operates at a fine vertical resolution of 10 cm and a spatial resolution of $0.5° \times 0.5°$, substantially

improving upon earlier models that either ignored spatial variability or applied coarse vertical layers. The model uses daily time steps, enhancing its sensitivity to climatic fluctuations and enabling the simulation of seasonal SIC patterns. Unlike previous models that used stochastic rainfall schemes, SINOCOM adopts deterministic rainfall inputs, reducing input uncertainty and improving predictive accuracy when driven by high-resolution climate datasets such as those from the CMIP6. This enables long-term carbon sequestration assessments across broad spatial and temporal scales under both historical and future climate scenarios. A key innovation of SINOCOM is the incorporation of soil thickness data to mechanistically link water fluxes with SIC transport [12]. This enables the model to accurately simulate both vertical and lateral SIC movement toward groundwater, addressing a critical limitation of previous models. Overall, SINOCOM provides an integrated, high-resolution platform that connects soil profile-scale carbonate processes with regional fluxes. Integrating SIC dynamics into Earth system models is essential for improving future predictions of soil carbon–climate feedbacks.

Supplementary Text 2: High spatial heterogeneity of SIC loss

The most pronounced losses were concentrated in central China, the Songnen Plain in northeastern China, and the southeastern and southern margins of the Tibetan Plateau. These regions are characterized by humid and dry sub-humid climates, where moisture availability accelerates carbonate dissolution and leaching (Fig. S5). The minimal SIC loss in southeastern China, which exhibits humid and dry sub-humid climate, were due to the widespread absence of SIC within the top 2 m of soil. This absence was attributed to long-term leaching under persistently humid conditions, which were shaped by the East Asian monsoon system in the east and the westerly-controlled climate of the inland basins in northwestern China. These conditions were established as early as the late Oligocene (approximately 24 million years ago) [13]. In addition, despite the sporadic occurrence of carbonate rocks in the region (Fig. S13), the flux of calcium carbonate in atmospheric dust was low [14], and the humid climate and dense vegetation further inhibited carbonate deposition from dust. This pattern coincided with areas characterized by low soil pH values [15,16]. Nevertheless, the residual SIC in southeastern China can be explained by localized carbonate rock weathering [17], the presence of carbonates in alluvial deposits [18,19], or anthropogenic contributions such as calcium carbonate enrichment in paddy soils resulting from prolonged cultivation [20]. SIC loss was observed along the southeastern margins of the Tibetan Plateau. This region, particularly the Hengduan Mountains, was influenced by both the South and East Asian summer monsoons, leading to high precipitation (Fig. S14) [21]. However, SIC accumulation persisted because of two main factors. First, an intensified monsoonal influence and increased precipitation occurred only after the uplift of the Tibetan Plateau, approximately 3–4 million years ago. This delayed the onset of intense leaching processes compared with that in eastern China [13,22]. Second, the Tibetan Plateau was among the world's most active dust sources, with high atmospheric inputs of calcium carbonate [14]. These factors, combined with extensive carbonate rock distributions (Fig. S13) and intense weathering processes, contributed to relatively high carbonate deposition fluxes and accumulation

rates in the region. In contrast, the northwestern regions of China, corresponding to hyper arid, arid, and semi-arid climatic regions, experienced minimal losses in SIC stocks. This pattern was strongly aligned with the national gradients of precipitation and evapotranspiration [23].

Supplementary Text 3: Methods

Environmental data

To project SIC dynamics under future climate conditions from 2015 to 2100, we employed climate projections from the Coupled Model Intercomparison Project Phase 6 (CMIP6). Four SSPs were selected to represent a range of possible socio-environmental futures: SSP1-2.6 (sustainability-focused development), SSP2-4.5 (intermediate socio-economic trajectory), SSP3-7.0 (regional rivalry and fragmentation), and SSP5-8.5 (fossil fuel-driven development) [24]. The climate model outputs were derived from the “r1i1p1f1” ensemble member and processed at a daily temporal resolution spanning from January 1, 2015, to December 31, 2100. Climate forcing variables were compiled from multiple CMIP6 models because no single model provides all the required environmental variables at daily time steps. Specifically, daily precipitation and air temperature data were obtained from the IPSL-CM6A-LR model at a spatial resolution of 250 km. Evapotranspiration estimates were derived from NorESM2-MM with a 100 km resolution. Monthly atmospheric CO_2 concentrations were obtained from Cheng et al. at a spatial resolution of 1°×1° [25]. Eight-day net primary productivity (NPP) data were employed estimate soil CO_2 concentrations [26]. All the variables were resampled to a spatial resolution of 0.5°×0.5°. Across all the SSP scenarios, the air temperature demonstrated a consistent upward trajectory throughout the simulation period, with the highest warming rate observed under SSP5-8.5, followed sequentially by SSP3-7.0, SSP2-4.5, and SSP1-2.6. The trend of atmospheric CO_2 concentrations was similar to that of temperature. The precipitation trends indicated a modest increase under SSP5-8.5 relative to those under the other pathways. Similarly, evapotranspiration exhibited a persistent increase over time (Fig. S8).

This study explicitly incorporated the effects of climate change, including the seasonal dynamics of soil CO_2, on SIC stocks. CO_2 is the primary driver of carbonate dissolution, originating from both atmospheric inputs and soil respiration. Accordingly, soil CO_2 concentrations were represented as a function of the atmospheric CO_2 concentration, temperature, and NPP, thereby capturing the combined influence of

climatic forcing and biotic carbon inputs on carbonate dynamics [27–29]:

$$pCO_{2(soil)} = pCO_{2(atm)} + \frac{A \cdot 0.75 \cdot NPP}{T^2}$$

where $pCO_{2(soil)}$ is the soil partial pressure of CO_2 (ppm), and $pCO_{2(atm)}$ is the partial pressure of CO_2 (ppm). CO_2 production in the rooting zone corresponds to 75% of the NPP [29]. The value of A used for the daily pCO_2 calculations is set to 3.7595×10^8 [28]. NPP represents the amount of dry biomass produced per unit area on a given day (g m^{-2} day^{-1}), and T denotes temperature (K).

Soil data

We compiled calcium carbonate content data from 2193 soil profiles containing carbonates down to 2 m, which were derived from China's national soil survey (Fig. S5) [30]. Additionally, we obtained volumetric water content data at 33 and 1500 kPa from SoilGrids, which represent the field capacity and wilting point, respectively. These datasets cover six standard soil depth intervals: 0–5, 5–15, 15–30, 30–60, 60–100, and 100–200 cm. Depth harmonization of the soil profile data was performed using an equal-area spline function [31], allowing the calculation of soil property values at a 10 cm resolution.

Given the well-established coupling between soil pH and the presence of calcium carbonate, i.e., a lack of carbonates at pH < 6.5, we excluded regions with pH values below this threshold within the 0–200 cm depth [15,16]. The pH data were collected from Chinese soil grids [12]. The spatial distribution of initial soil inorganic carbon density (SICD) was subsequently modeled using digital soil mapping techniques under the assumption that soil variability can be expressed as a function of soil-forming factors, such as climate and topography. A random forest algorithm was employed to generate national-scale SICD maps based on the soil profile dataset in Soil Series of China [32], providing a baseline SIC distribution for the year 2015 (Fig. S1 a–d). The output map was resampled to a spatial resolution of 0.5°×0.5° and then clipped using high-resolution soil thickness data to retain layer-specific soil depth information [12]. These processed raster datasets served as inputs to the SIC turnover model for

simulating future SIC dynamics under climate change.

Model assumptions

To isolate the response of SIC dynamics to climate change while ensuring computational efficiency, the model incorporates the following simplified assumptions. (1) In addition to climate change, the dynamics of SIC are influenced by external acid inputs, such as those from acid deposition and anthropogenic activities [15]. However, this study focuses specifically on the natural dissolution processes of SIC under climate-driven conditions, thereby assuming no significant contributions from external acid sources within the soil system. (2) Water was assumed to move through the soil column as piston flow, neglecting unsaturated flow and nonuniform wetting fronts [33]. (3) Given the uncertainties in accurately distinguishing limestone from the less abundant dolostone in existing geological maps, we assumed that all carbonate outcrops consisted of calcite in this study [10,34]. (4) Calcite kinetics in soils were generally considered rapid such that dissolution and precipitation could be treated as instantaneous processes governed by thermodynamic equilibrium. Under this assumption, mineral–solution interactions were represented through equilibrium constants, and potential rate limitations were not explicitly incorporated. This treatment reduced model complexity and reflected the prevailing view that carbonate minerals respond quickly to shifts in carbonate chemistry [5,10,33]. (5) The model assumed no calcium release from silicate mineral weathering, with Ca^{2+} sourced exclusively from calcite. (6) It was assumed that microbial activities had no direct influence on calcite precipitation within the soil system [10]. These assumptions, while idealized, facilitated a tractable representation of SIC turnover under the projected climate scenarios and provided a focused lens on the abiotic controls governing carbonate dynamics in natural soil systems.

Model structure

We developed a new process-based SIC turnover model (SINOCOM) to simulate SIC dynamics, which integrates two primary sub-models: a water balance module and a carbonate chemical equilibrium module (Fig. 1). The model explicitly accounts for

changes in soil water content induced by precipitation and evapotranspiration, which drive vertical water movement across soil layers. The SIC in each soil layer undergoes chemical reactions, which are modulated by variations in temperature, soil CO_2 concentration, and soil moisture. During these processes, dissolved chemical species are transported with percolating water, coupling hydrological fluxes with SIC chemical transformations. The simulations were conducted at a daily temporal resolution and a vertical resolution of 10 cm per soil layer, enabling detailed tracking of SIC dynamics under varying environmental conditions.

Water balance model of SIC leaching and deposition

The processes of SIC dissolution, leaching, and re-precipitation occur within the soil water environment. One-dimensional compartmentalized models divided the soil column into 10 cm compartments, each with initially specified properties and distinct environmental conditions [5,10,33]. These properties regulated both the local soil water balance and the equilibrium processes governing $CaCO_3$ dissolution and precipitation. Soil moisture is bounded by field capacity, defined as the water content remaining after saturation and free drainage and the permanent wilting point at which plants cannot recover even if water becomes available [35]. At the beginning of each simulation time step, rainfall initially infiltrated the topsoil [10]. Once the volumetric water content in the top layer exceeded its field capacity, the excess water was redistributed to the underlying layer [35]. Evapotranspiration removed moisture from the upper layers. Therefore, when the water content in the surface soil dropped below the wilting point, water from the lower compartments was drawn upward via capillary rise to meet the demand for evapotranspiration. This process continued until either the demand was fulfilled or all the plant-available water was depleted. Therefore, if the soil moisture exceeded field capacity, it was reset to soil moisture, resulting in a net increase in soil water, whereas net infiltration was assumed to be zero if the soil moisture was below field capacity [35]. Once the soil water reached the bottom of the profile defined by a limited soil depth [12], the remaining water was lost through deep drainage or subsurface flow to the lateral groundwater system [36–38]. Soil water characterization

involved parameters like such as field capacity (volumetric water content data at 33 kPa), wilting point (volumetric water content data at 1500 kPa), water holding capacity and initial soil moisture conditions derived from SoilGrids (Table S2).

Chemical equilibrium model for SIC dissolution

SIC comprises solid-phase carbonate minerals, gaseous CO_2 in soil, and dissolved inorganic carbon in soil solution. The dominant component of SIC is solid-phase $CaCO_3$; therefore, the primary turnover processes of SIC are governed by geochemical equilibrium within the $CaCO_3$-CO_2-H_2O system. In this study, we quantified the chemical reactions of calcium carbonate using thermodynamic principles. To ensure accurate representation of these processes, we systematically reviewed the literature, collating and comparing quantitative calculation methods and processes for calcium carbonate chemical reactions derived from extensive field-based chemical monitoring and laboratory-based solution chemistry experiments. Through this comparative analysis, we identified the key chemical reactions and parameters that control carbonate system dynamics in soils. All the equilibrium constants and their temperature dependencies employed in this study were carefully evaluated and validated [11] (Supplementary Text 1; Table S1).

We additionally quantified pedogenic carbonate formation from atmospheric CO_2. We first simulated SIC dynamics under the assumption of no external Ca^{2+} inputs, thereby estimating the total SIC loss driven solely by in situ carbonate dissolution. We then incorporated environmental Ca^{2+} sources by compiling province-level Ca^{2+} concentrations in precipitation across China (Tables S10, 11). During days with rainfall, Ca^{2+} input from precipitation was added to the soil water- balance module to calculate the Ca^{2+} concentrations in the soil solution, which were subsequently passed to the carbonate chemistry module. Under these conditions, the model simulated both CO_2-driven pedogenic carbonate formation during drying cycles and net SIC loss resulting from dissolution–precipitation processes. The difference between simulations with and without an external Ca^{2+} supply represented pedogenic carbonate formation from CO_2 under drying conditions (Table S4).

In the $CaCO_3$-CO_2-H_2O system, calcite surfaces experience variable degrees of chemical attack by H_2O-CO_2 solutions.

$$CaCO_3 + H_2O \rightleftharpoons Ca^{2+} + CO_3^{2-} + H_2O \tag{1}$$

$$CaCO_3 + H_2CO_3 \rightleftharpoons Ca^{2+} + 2HCO_3^{-} \tag{2}$$

The reaction between the H_2O-CO_2 system and $CaCO_3$ was regulated by the concentrations of H^+, HCO_3^-, CO_3^{2-}, H_2CO_3, and Ca^{2+} at the calcite surface. To clarify the chemical equilibrium among these species, the $CaCO_3$-H_2O-CO_2 system involved the following reactions [11]:

Gaseous CO_2 is dissolved into the aqueous phase as follows:

$$CO_2^{g} + H_2O \rightleftharpoons CO_2^{aq} + H_2O \tag{3}$$

$$K_D = \frac{(CO_2^{aq})}{(CO_2^{g})} \tag{4}$$

where K_D is the partition coefficient of CO_2 between the gaseous and aqueous phases.

As shown in Table S2, according to Henry's law, the partial pressure of CO_2 (pCO_2) is related to the activity of dissolved CO_2:

$$(CO_2^{aq}) = K_H \cdot p_{CO_2} \tag{5}$$

where K_H is Henry's constant depended on temperature.

Parentheses denote activity, square brackets denote concentration, and the activity coefficient (γ) depends on solute concentration. K_H and K_D are related by the following:

$$K_D = K_H \cdot R \cdot T \tag{6}$$

where T is the absolute temperature and R is the gas constant. CO_2 reacts with water to form carbonic acid:

$$H_2O + CO_2 \rightleftharpoons H_2CO_3 \tag{7}$$

$$(CO_2^{aq}) = K_0 \cdot (H_2CO_3) \tag{8}$$

where K_0 is the equilibrium constant for this reaction. It is typically defined as follows:

$$[H_2CO_3^{*}] = [CO_2^{aq}] + [H_2CO_3] \tag{9}$$

$$\frac{(CO_2^{aq})}{(H_2CO_3^*)}=\left(1+\frac{1}{K_0}\right)^{-1} \tag{10}$$

H_2CO_3 dissociates into H^+ and HCO_3^-:

$$H_2CO_3 \rightleftharpoons H^+ + HCO_3^- \tag{11}$$

$$\frac{(H^+)(HCO_3^-)}{(H_2CO_3^*)}=K_1 \text{ or } \frac{(H^+)(HCO_3^-)}{(H_2CO_3)}=K_1\cdot(1+K_0)=K_{H_2CO_3} \tag{12}$$

where K_1 is the dissociation constant of H_2CO_3.

The second dissociation step is as follows:

$$HCO_3^- \rightleftharpoons H^+ + CO_3^{2-} \tag{13}$$

$$\frac{(H^+)(CO_3^{2-})}{(HCO_3^-)}=K_2 \tag{14}$$

where K_2 is the dissociation constant of HCO_3^-.

Finally, the dissociation of water occurs as follows:

$$(H^+)(OH^-)=K_W \tag{15}$$

where K_W is the dissociation constant of H_2O.

The law of mass action is expressed in terms of activity, which is related to concentration via the activity coefficient. The activity coefficient is influenced by the ionic strength (I) of the solution:

$$I=\frac{1}{2}\sum_i Z_i^2\cdot c_i \tag{16}$$

where Z_i is the charge of ion i in solution and c_i is the concentration of ion i. For CO_2 dissolved in pure water:

$$I=\frac{1}{2}\left([H^+]+[OH^-]+[HCO_3^-]+4[CO_3^{2-}]\right) \tag{17}$$

Activity and concentration are interrelated:

$$(i)=\gamma_i\cdot[i] \tag{18}$$

where γ_i is the activity coefficient. (i) denotes activity, and [i] denotes concentration. The activity coefficient for individual ions is typically calculated using the equation as

following equation:

$$\log \gamma_i = -Az_i^2 \cdot \frac{\sqrt{I}}{1 + Ba_i\sqrt{I}} + b_i I \tag{19}$$

where A and B are parameters of the ion activity coefficients that depend on temperature. a and b are ionic coefficients. If $CaCO_3$ dissolves, the equilibrium between the dissolved species and the solid is defined by the solubility product K_C as follows:

$$K_c = (Ca^{2+})_{eq}(CO_3^{2-})_{eq} \tag{20}$$

where K_C is the solubility product of $CaCO_3$ and $(Ca^{2+})_{eq}$ and $(CO_3^{2-})_{eq}$ represent the equilibrium activities of Ca^{2+} and CO_3^{2-}, respectively. The saturation state of the solution can be described using the saturation index as follows:

$$\Omega = \frac{(Ca^{2+})(CO_3^{2-})}{K_c} \tag{21}$$

when $\Omega < 1$, the solution is undersaturated; when $\Omega = 1$, it is saturated; and when $\Omega > 1$, it is supersaturated.

Boundary conditions for achieving equilibrium

The soil system is conceptualized as a three-phase open system comprising solid, liquid, and gaseous components, wherein gaseous exchange occurs continuously between soil air and the overlying atmosphere. Within this framework, the volume of solution in contact with $CaCO_3$ was denoted as V_l [11], while the volume of the gaseous phase in contact with CO_2 was denoted as V_g. Given the soil–atmosphere interface and the assumption of continuous gas exchange, the model treated the soil system as an open system, allowing water to dissolve $CaCO_3$ under conditions analogous to those of a free water surface. Accordingly, the gas-phase volume was considered effectively infinite ($V_g \rightarrow \infty$), and the ratio Vg/V_l was assumed to be 10^5. However, the dissolved bicarbonate (HCO_3^-) subsequently precipitated as carbonate in soil water or groundwater. The dissolution of $CaCO_3$ was assumed to occur slowly enough that equilibrium was maintained among all carbonate species in the solution, gas, and solid phases. Using equilibrium constants, ion activities, acid–base equilibria, and defined

boundary conditions at equilibrium, we derived a set of equations to calculate the concentrations of major ionic species involved in the $CaCO_3$-CO_2-H_2O system [11,39].

The dissolution of each mole of $CaCO_3$ releases one mole of Ca^{2+} and one mole of carbonate species into the soil solution. Consequently, the total carbon content within the system is governed by the conservation of carbonate species, which reflects the conservation of carbon atoms:

$$\begin{aligned} M_T &= \left([Ca^{2+}]+[HCO_3^-]_i+[H_2CO_3^*]_i+[CO_3^{2-}]_i\right)\cdot V_1+[CO_2^g]_i V_g \\ &= \left([HCO_3^-]+[H_2CO_3^*]+[CO_3^{2-}]_i\right)\cdot V_1+[CO_2^g]V_g \end{aligned} \tag{22}$$

where M_T is the total carbon content within the system.

The numerical framework of SINOCOM is built upon established thermodynamic chemical reactions. By solving for the equilibrium concentrations of key ionic species, including Ca^{2+}, HCO_3^- and CO_3^{2-}, the model quantifies changes in $CaCO_3$ concentration at each time step based on the difference between the initial Ca^{2+} concentration in the soil solution and its equilibrium concentration. The model specifically targets the equilibrium state of the soil carbonate system. Given the initial soil CO_2 partial pressure and the initial Ca^{2+} concentration in the soil solution, the complete chemical speciation of the system is iteratively determined (the detailed computational procedures follow the thermodynamic approach described by Dreybrodt [11,39]):

$$[H^+]=-\frac{1}{2}(2[Ca^{2+}]-[HCO_3^-])+\left\{\frac{1}{4}(2[Ca^{2+}]-[HCO_3^-])^2+\frac{K_W}{\gamma_H\gamma_{OH}}+\frac{2K_2\gamma_{HCO_3}}{\gamma_H\gamma_{CO_3}}[HCO_3^-]\right\}^{1/2} \tag{23}$$

$$[HCO_3^-]=\frac{[HCO_3^*]K_1}{\gamma_H\gamma_{CO_3}[H^+]}=\frac{(1+K_0^{-1})P_{CO_2}K_H K_1}{\gamma_H\gamma_{CO_3}[H^+]} \tag{24}$$

$$[CO_3^{2-}]=\frac{\gamma_{HCO_3}[HCO_3^-]K_2}{\gamma_H[H^+]}=\frac{\gamma_{HCO_3}(1+K_0^{-1})P_{CO_2}K_H K_1 K_2}{\gamma_H^2\gamma_{CO_3}[H^+]^2} \tag{25}$$

$$[OH^-]=\frac{K_W}{\gamma_H\gamma_{OH}}\frac{1}{[H^+]} \tag{26}$$

Sensitivity analyses

We employed two complementary approaches to assess model uncertainty. The first approach involved a sensitivity analysis to quantify the influence of climatic input variability on model outputs and to identify key parameters driving SIC dynamics. Specifically, we conducted a local sensitivity analysis using a one-at-a-time (OAT) approach [40,41], whereby each parameter was perturbed individually while all the others were held constant. This approach allowed us to systematically evaluate the long-term sensitivity of SIC to variations in climatic drivers. We tested the response of the SIC to ±5% and ±10% changes in primary climatic variables: precipitation, temperature, evapotranspiration, CO_2 concentration and NPP. The magnitude of SIC change resulting from each perturbation was calculated, and the absolute rate of SIC change was used to indicate the relative sensitivity and importance of each parameter in controlling SIC turnover [40].

The second approach focused on evaluating uncertainties associated with the chemical equilibrium constants that govern $CaCO_3$ reactions. We distinguished the uncertainties of the water balance model and the carbonate chemical balance model. In the water balance model, we selected three key parameters: the wilting point, water holding capacity, and initial soil moisture conditions. In the chemical equilibrium model, we selected five key thermodynamic parameters: Henry's law constant (K_H), the dissociation constant of H_2CO_3 (K_1), the dissociation constant of HCO_3^- (K_2), the dissociation constant of water (K_W), and the solubility product of calcium carbonate (K_C). To quantify how uncertainties in these constants propagated through the model, we conducted a Monte Carlo simulation by specifying plausible parameter ranges for each constant. The primary sources of uncertainty included spatial heterogeneity in atmospheric pressure and methodological differences in deriving equilibrium constants from empirical data. Given the limited research on the spatial variability and fluctuation ranges of these constants, we assigned coefficients of variation (CVs) based on a qualitative assessment of data reliability. In the absence of truly independent datasets, a CV of 0.1 was assigned to parameters associated with highly reliable constants,

reflecting their relatively low uncertainty [42]. We further assumed that the input parameters were statistically independent, allowing them to be sampled separately from their respective distributions. The model was run for 100 Monte Carlo iterations by randomly sampling input values from the prescribed distributions, with model uncertainty quantified by the standard deviation of the outputs.

Validation

We searched for articles across Web of Science, Google Scholar, and CNKI (China National Knowledge Infrastructure) for studies published between January 2015 and December 2024 using the keywords "soil inorganic carbon/soil inorganic carbon stock/soil inorganic carbon dissolution/soil inorganic carbon density/soil inorganic carbon cycling." Studies were included only if soil sampling was performed between 2015 and 2024 and samples were collected from natural ecosystems to minimize anthropogenic disturbance to SIC dynamics. A total of 36 studies met these criteria (Table S12), with 133 sampling sites (Fig. S15) and 593 individual observations. Sampling years and geographic coordinates were used to extract corresponding SIC outputs from the SINOCOM simulations, enabling model performance evaluation. Additionally, site-level observations were aggregated to estimate annual SIC changes for each grid cell to further validate the modeled SIC dynamics.

In model applications, parameters that are directly measurable or empirically constrained, such as soil hydraulic properties, including initial soil moisture conditions, field capacity, and wilting point, should be assigned values that are consistent with observations. Parameters for which fixed values cannot be empirically determined may be derived from the literature of the study area. This applies, for example, to chemical equilibrium constants K_H, K_1, K_2, K_W, and K_C. After model simulations, parameter estimation and uncertainty analysis should be conducted by defining credible confidence bounds for each parameter, based on physical constraints or experimental evidence. This approach enables the quantification of parameter-induced uncertainty in model outputs. Sensitivity analysis can then be performed to identify those most critical to simulations of the SIC cycle.

Combination of machine learning and process-based models

Process-based models are well suited for resolving the dominant mechanisms operating within minor soil profiles, yet their spatial deployment is often constrained by limited data as well as high computational demand. In contrast, machine- learning (ML) approaches excel at spatial prediction but do not explicitly represent soil processes. To leverage the strengths of both frameworks, we integrated the process-based SINOCOM model with ML algorithms, thereby enhancing our ability to simulate SIC dynamics consistently across space and time [43]. One effective strategy is to use outputs from the process-based model as supplementary spatiotemporal training data for ML models. Here, we assembled 0.5° resolution datasets of climate variables, soil properties, and SINOCOM-simulated SIC for four depth intervals (0–10, 0–30, 0–100, and 0–200 cm) covering 2020–2100. Using these data, we conducted a systematic ML preassessment and compared the predictive skill of four algorithms—random forest (RF), extreme gradient boosting (XGBoost), and support vector regression (SVR). For each depth, the dataset was randomly partitioned into training and testing subsets (8:2), and model performance was evaluated using the coefficient of determination (R^2). RF consistently achieved the highest R^2 across all depth intervals (Table S5) and was thus selected as the primary algorithm. Based on RF, we developed 36 independent predictive models (four depths × nine decadal time steps from 2020 to 2100) and assessed their robustness using R^2 (Table S6). We then combined the trained models with 1-km digital soil maps of SIC density and corresponding climate and soil covariates to generate national-scale, 1-km SIC datasets for each decade and soil layer for 2020 to 2100 (Fig. S1). Ultimately, we provide annual SIC density outputs from the process-based model at 0.5° resolution for 0–10, 0–30, 0–100, and 0–200 cm soil layers and decadal ML-downscaled SIC density dataset at 1-km resolution for the same soil depth intervals (https://figshare.com/s/94d57494e4748107516e).

Supplementary Figures

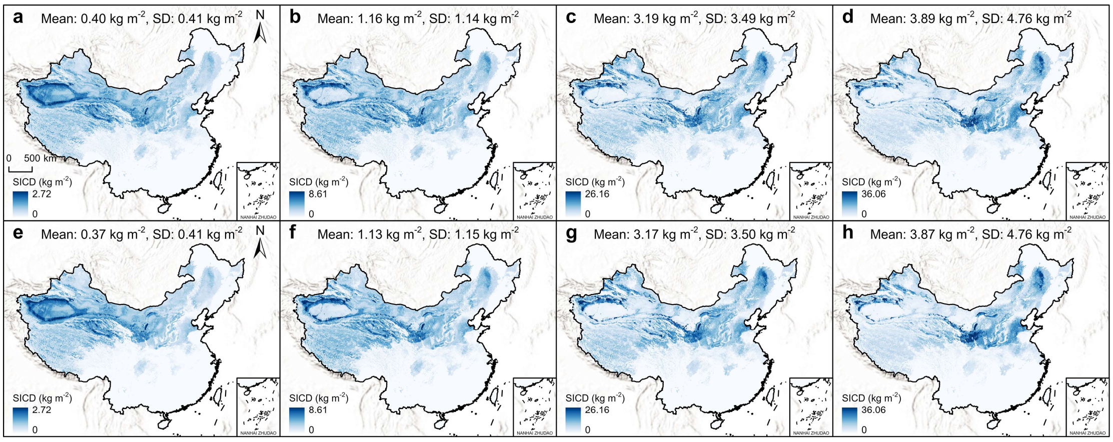

审图号: GS京(2026)0201号

Figure S1. Spatial distribution of SIC across soil depths under climate change in 2015 and 2100 at a 1-km spatial resolution. **a–d** Spatial distribution of SICD for the 0–10 cm (a), 0–30 cm (b), 0–100 cm (c), and 0–200 cm (d) soil layers in 2015. **e–h** Spatial distribution of SICD corresponding to the same groupings as in a–d in 2100.

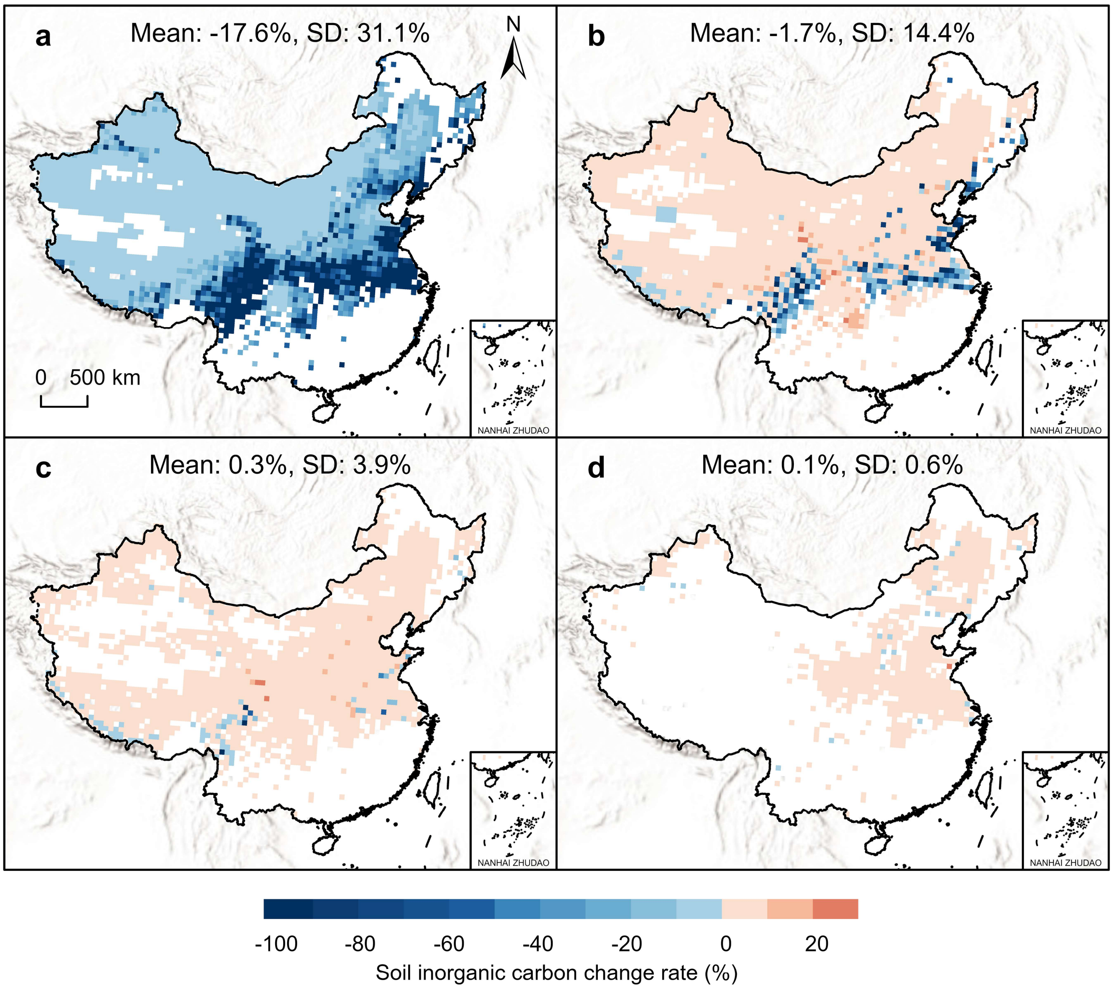

审图号: GS京(2026)0201号

Figure S2. Spatial distribution of the SIC change rate across soil depths under climate change from 2015 to 2100. a–d Spatial distribution of the SIC change rate under SSP2-4.5 for the 0–10 cm (a), 10–30 cm (b), 30–100 cm (c), and 100–200 cm (d) soil layers.

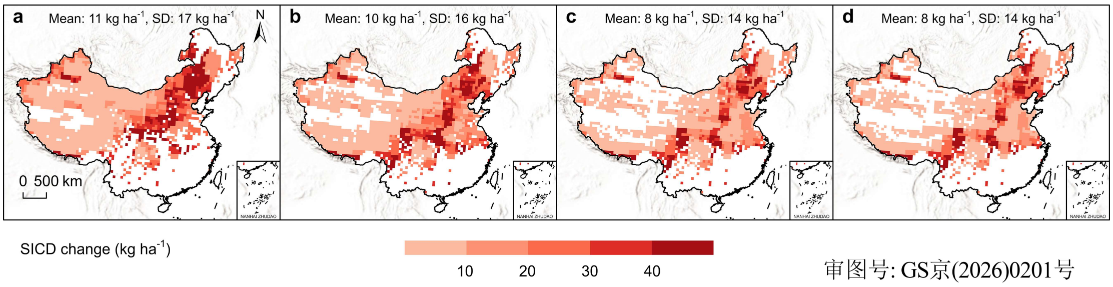

Figure S3. Spatial distribution of new formation of SIC across soil depths under climate change in 2015 and 2100. **a–d** Spatial distribution of new formation of SICD in the 0–10 cm (a), 0–30 cm (b), 0–100 cm (c), and 0–200 cm (d) soil layers.

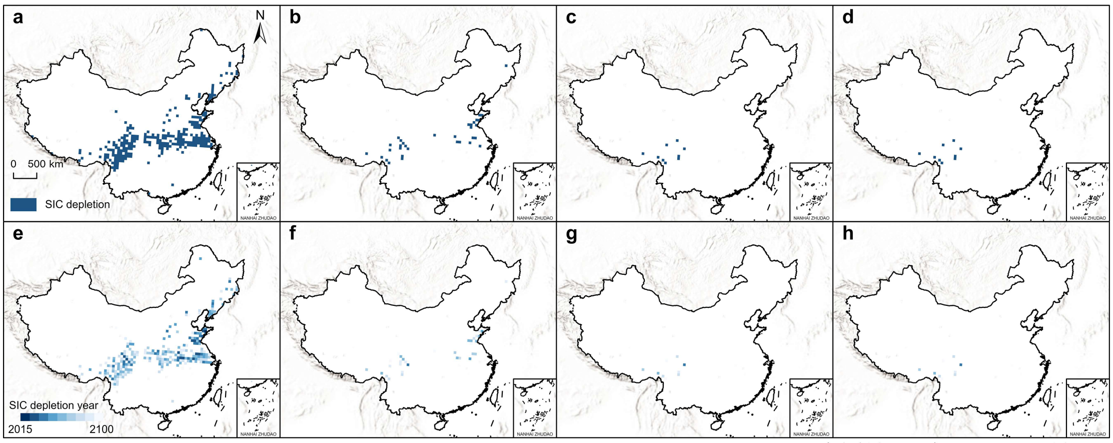

审图号: GS京(2026)0201号

Figure S4. Spatial distribution of SIC depletion across soil depths under climate change for 2015 to 2100. **a–d** SIC depletion areas for the 0–10 cm (a), 0–30 cm (b), 0–100 cm (c), and 0–200 cm (d) soil layer. **e–h** SIC depletion year corresponding to the same groupings as in a–d, respectively.

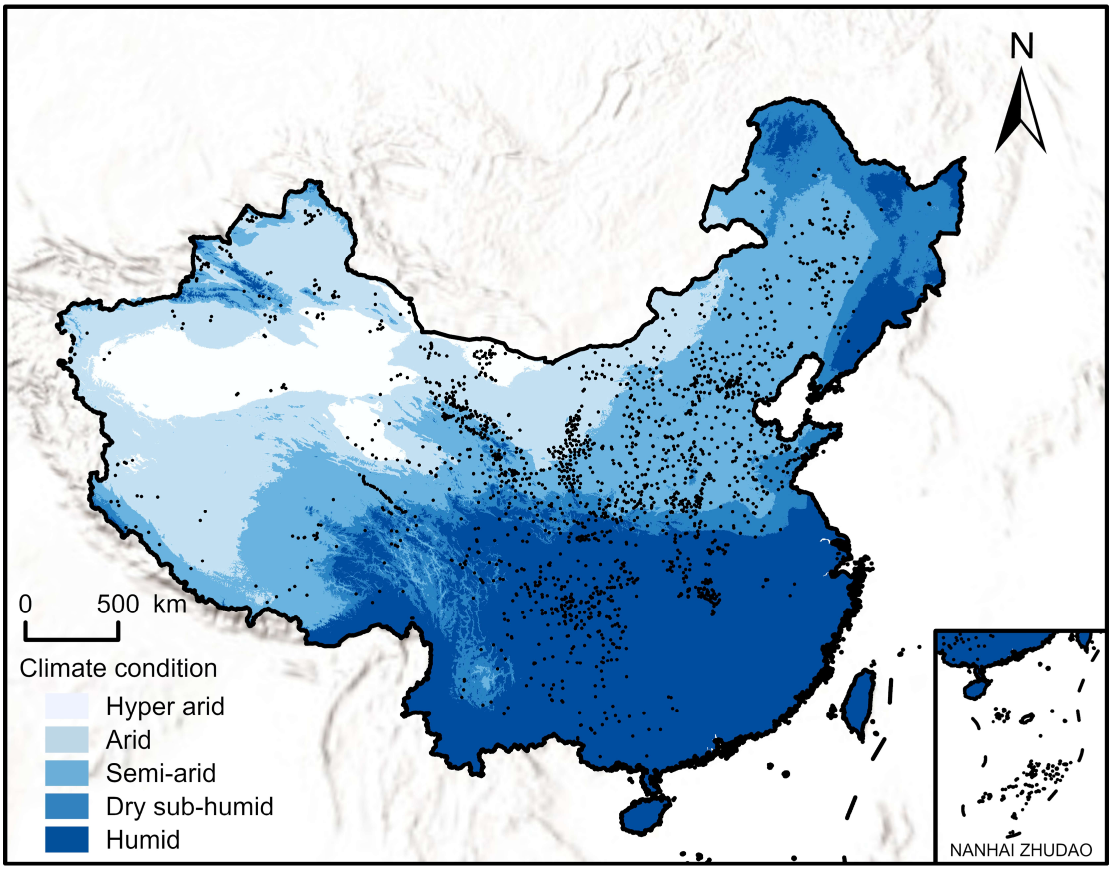

审图号: GS京(2026)0201号

Figure S5. Distribution of 2,193 soil profiles containing SIC in this research. Climatic conditions were defined by the aridity index (AI) as follows: hyper arid (AI < 0.03), arid (0.03 < AI < 0.2), semi-arid (0.2 < AI < 0.5), dry sub-humid (0.5 < AI < 0.65), and humid (AI > 0.65) [44]. Soil samples were derived from the Soil Series of China [30].

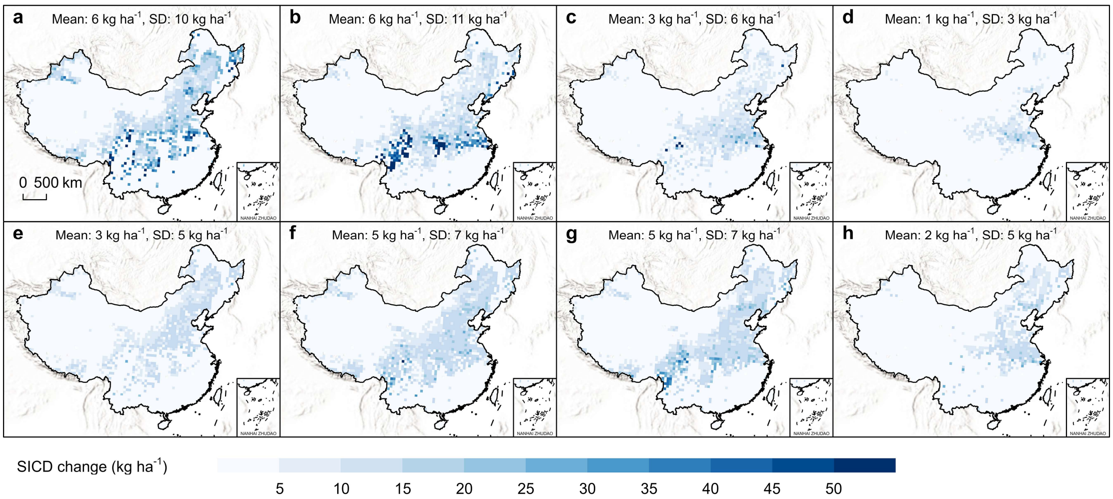

审图号: GS京(2026)0201号

Figure S6. Standard deviations of the changes in SIC based on 100 simulations. a–d Standard deviations of the change in SICD of the water balance module for the 0–10 cm (a), 10–30 cm (b), 30–100 cm (c), and 100–200 cm (d) soil layers. **e–h** Standard deviations of the change in SICD of the carbonate chemical equilibrium module corresponding to the same groupings as in a–d in 2100.

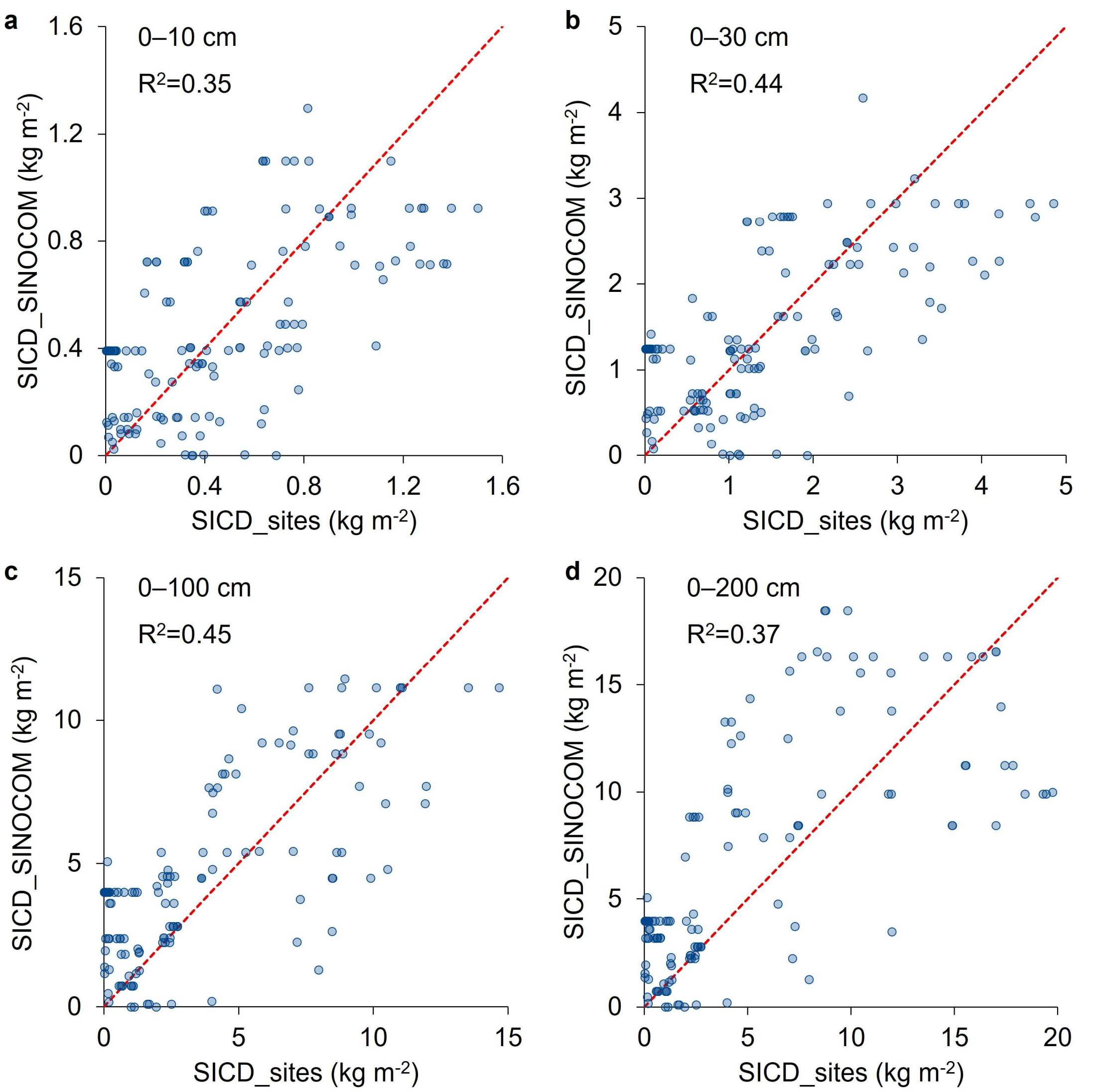

Figure S7. Observed versus predicted SICD for samples where SICD values were validated. a–d Observed versus predicted SICD for the 0–10 cm (a), 0–30 cm (b), 0–100 cm (c), and 0–200 cm (d) soil layers. The red line indicates the 1:1 line.

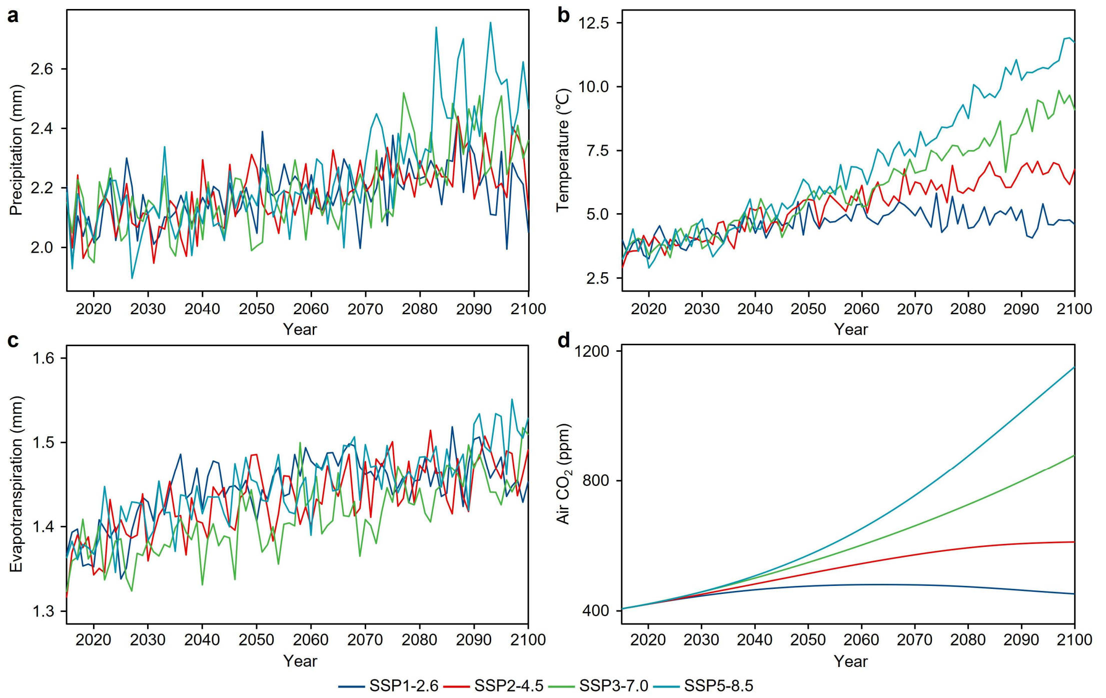

Figure S8. Long-term climate trends under four climate scenarios for 2015 to 2100. **a–d** Annual mean changes in precipitation (a), temperature (b), evapotranspiration (c), and air CO_2 concentration (d) across China.

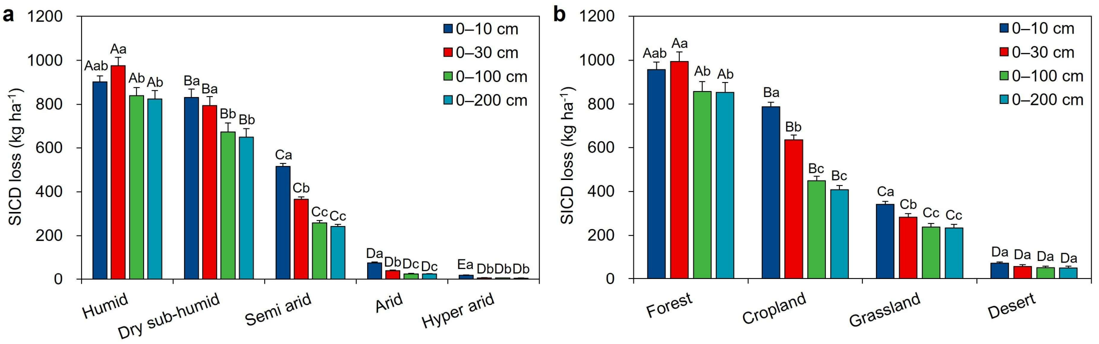

Figure S9. SIC loss under climate change from 2015 to 2100. a–b SICD loss by soil depth under different climatic conditions (a) and land use (b). The error bars indicate the standard errors across China. The capital letters indicate significant differences among climatic conditions (a) and land use (b) ($p < 0.05$). The small letters to indicate significant differences between different soil depths ($p < 0.05$).

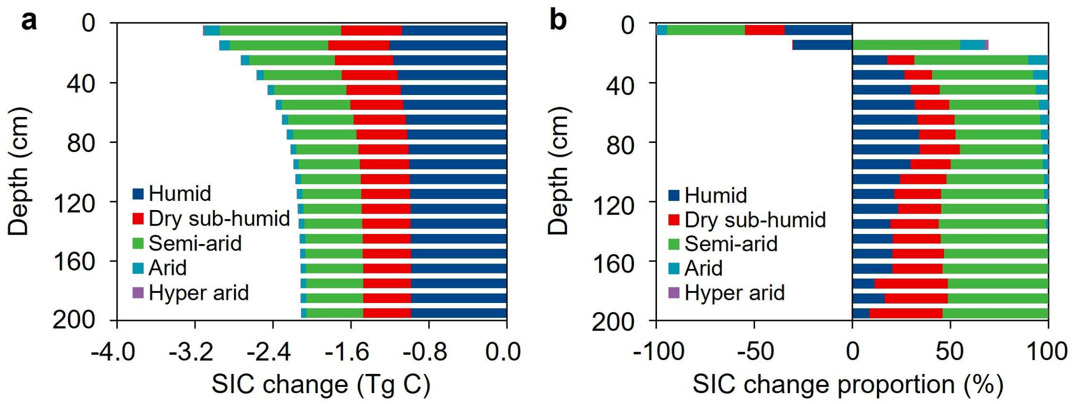

Figure S10. SIC leaching and accumulation patterns in soil profiles from 2015 to 2100. a–b Total cumulative (a) and absolute proportional (b) changes in SIC stocks across depths under different climate conditions.

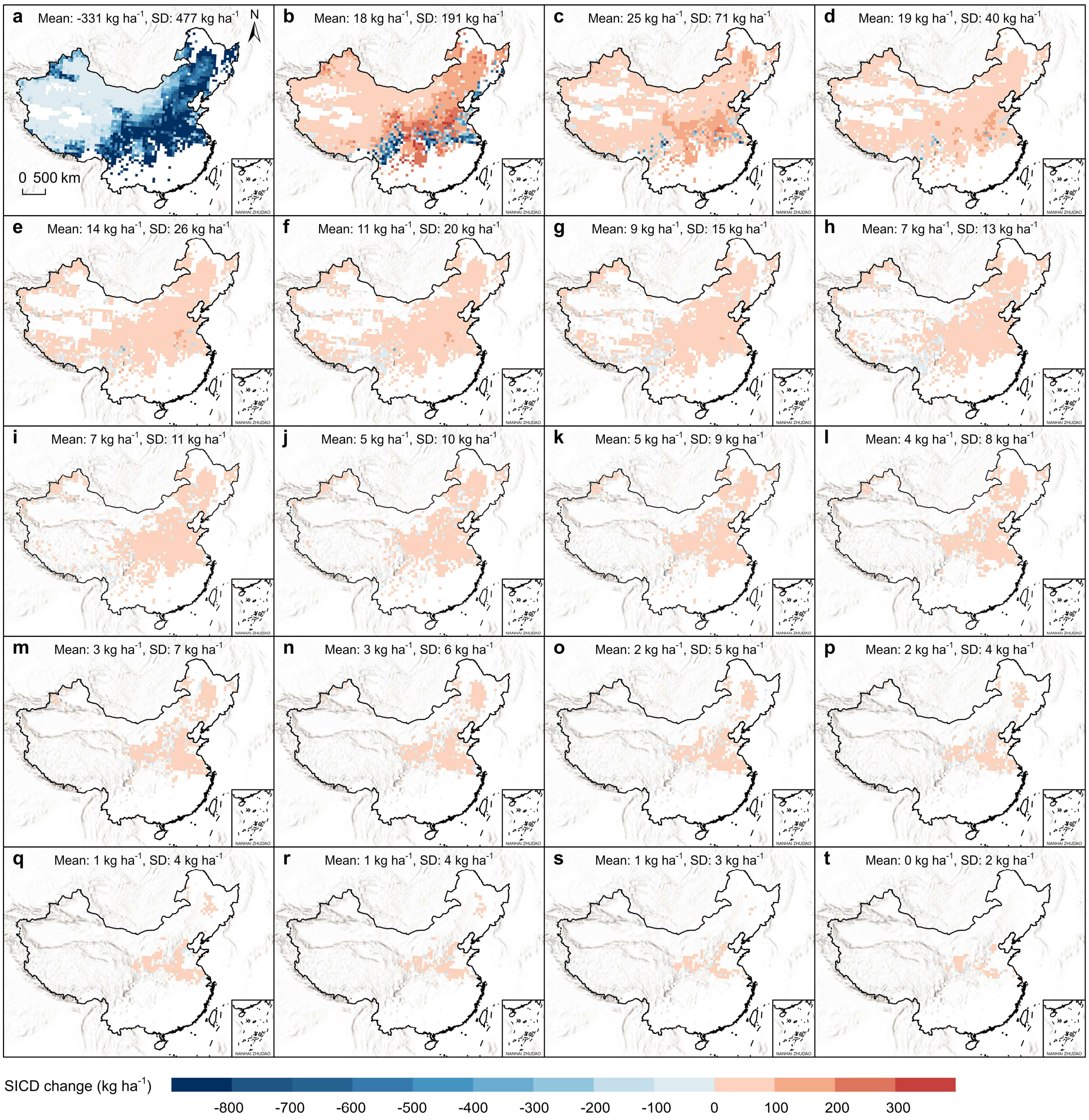

审图号: GS京(2026)0201号

Figure S11. SIC leaching and accumulation in soil profiles from 2015 to 2100 at 10 cm depth intervals. a–t SIC leaching and accumulation for the 0–10 cm (a), 10–20 cm (b), 20–30 cm (c), 30–40 cm (d), 40–50 cm (e), 50–60 cm (f), and 60–70 cm (g), 70–80 cm (h), 80–90 cm (i), and 90–100 cm (j), 100–110 cm (k), 110–120 cm (l), and 120–130 cm (m), 130–140 cm (n), 140–150 cm (o), and 150–160 cm (p), 160–170 cm (q), 170–180 cm (r), 180–190 cm (s), and 190–200 cm (t) soil layers.

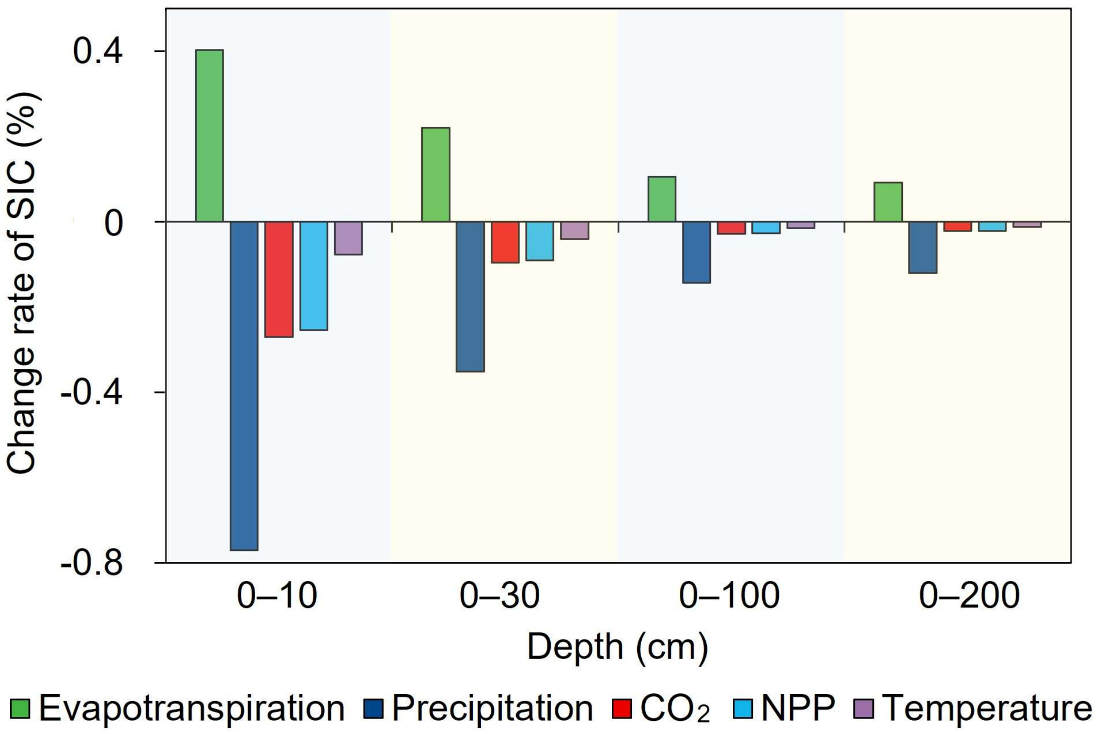

Figure S12. Parameter sensitivity for SIC turnover across soil depths. SIC response to a 10% increase in model parameters across depth intervals.

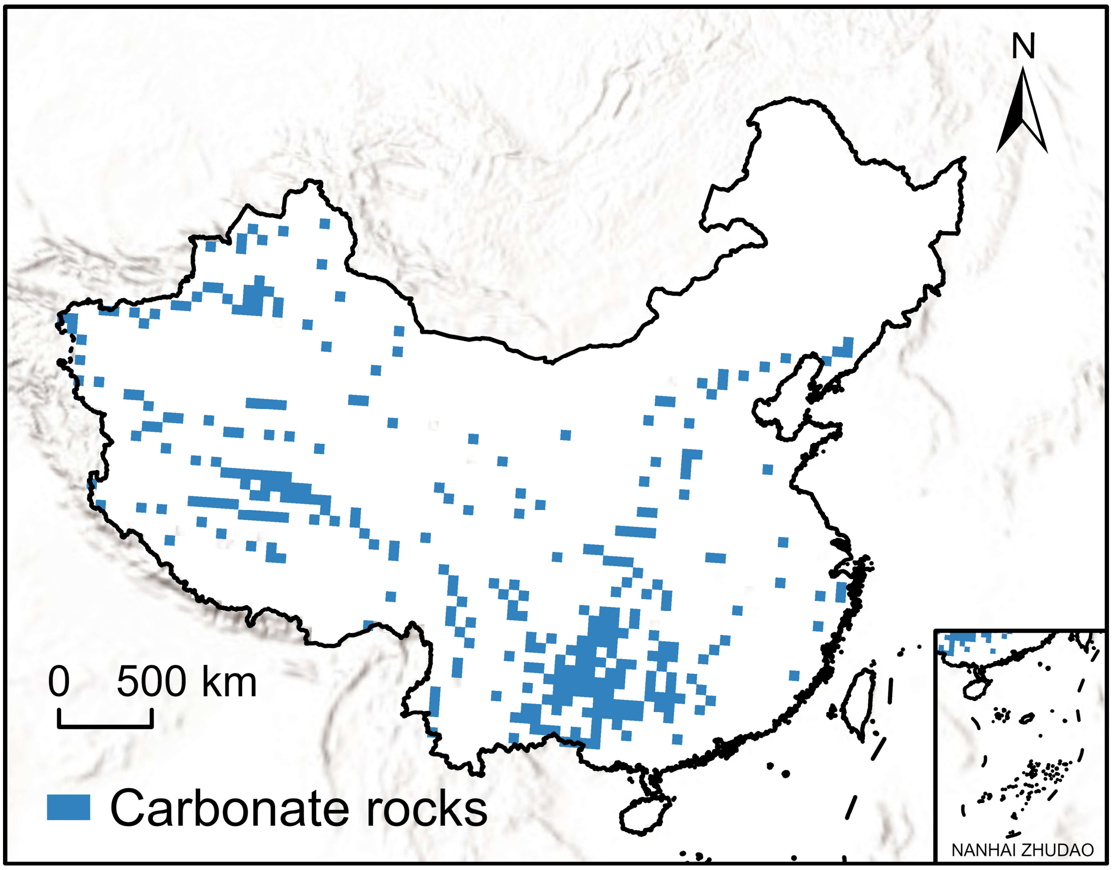

审图号: GS京(2026)0201号

Figure S13. Distribution of carbonate rocks in China [17].

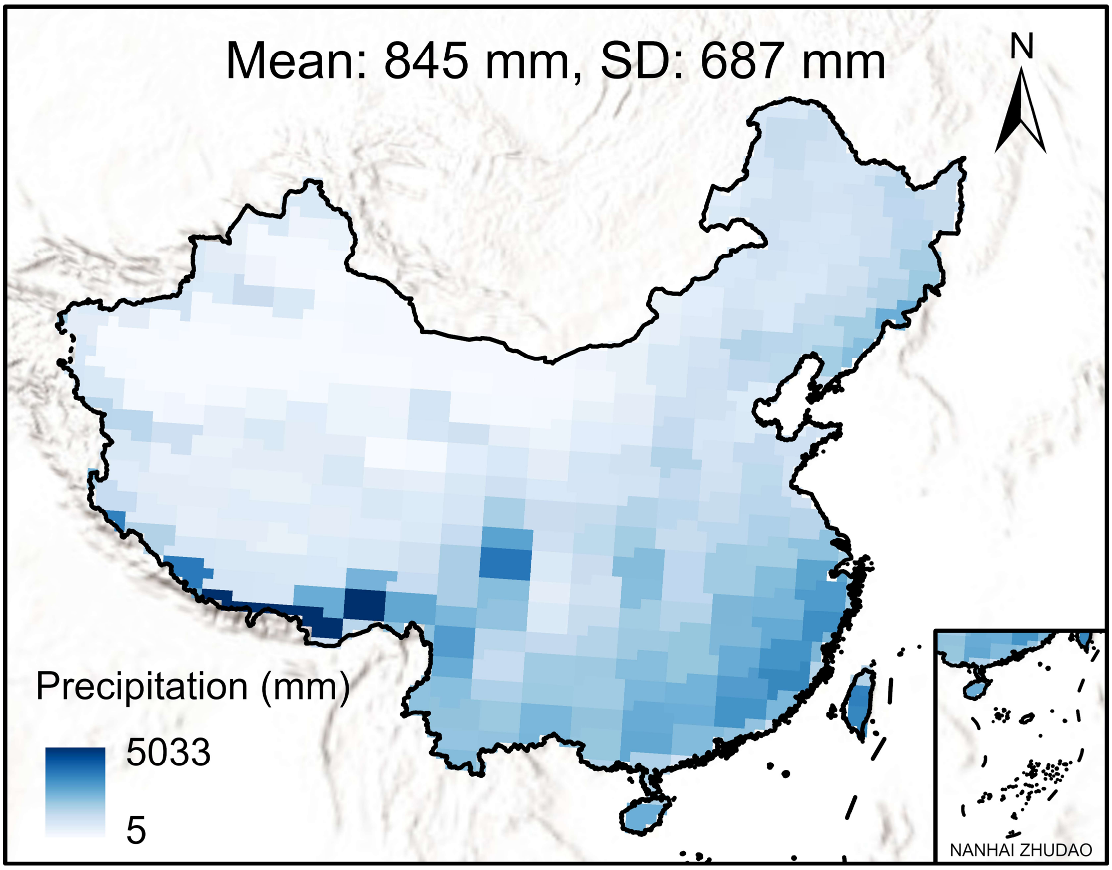

审图号: GS京(2026)0201号

Figure S14. Mean annual precipitation distribution map for 2015–2100 under SSP2-4.5 in China.

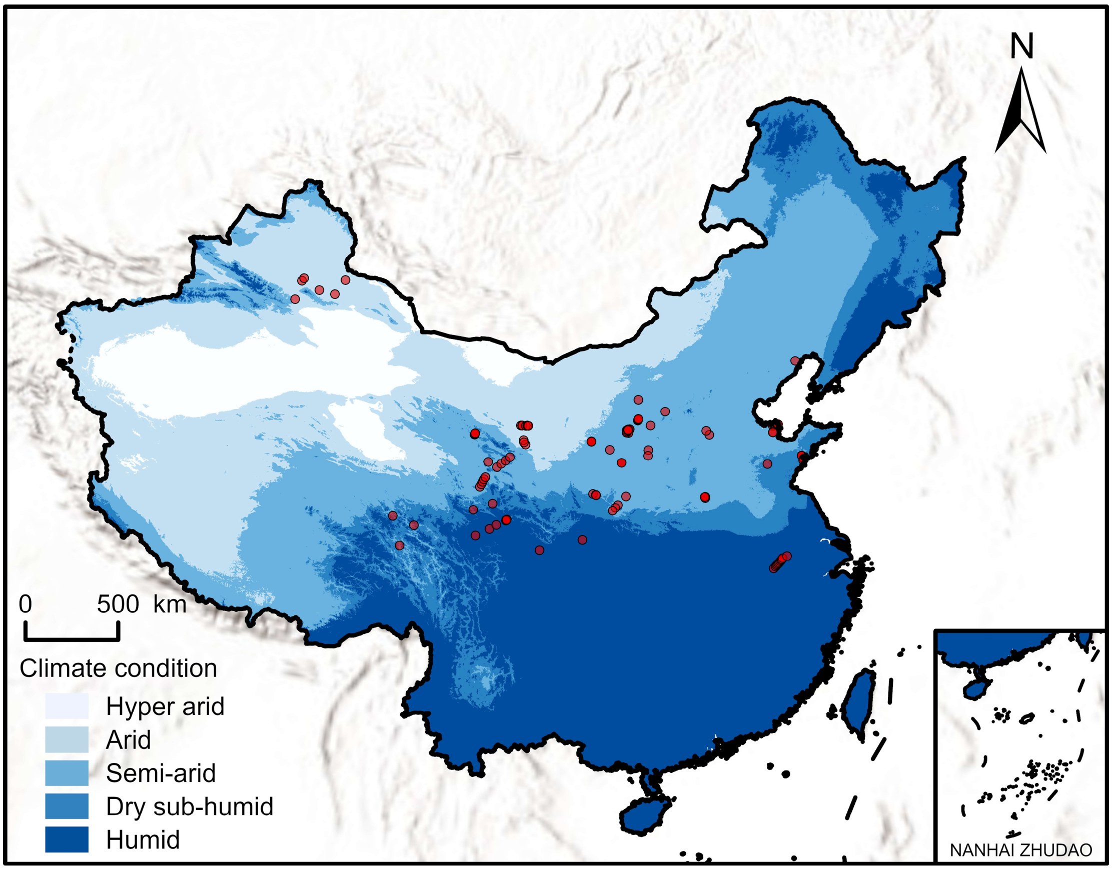

审图号: GS京(2026)0201号

Figure S15. Distribution of 133 soil profiles containing SIC used for validation in this research.

Supplementary Tables

Table S1. Comparative review of existing SIC models.

Reference	Publish year	Model name	Form mechanism	Ca^{2+} source	Chemistry	Vertical resolution	Time resolution	Spatial resolution	Land hydrology	Groundwater output	Other Minerals
This study	2025	SINOCOM	Illuvial	Precipitation	Thermodynamic equilibrium, acid base iteration, iterative process of carbonate dissolution and saturation	10 cm	Daily	0.5°	Yes	Yes	No
Arkley[1]	1963		Illuvial	Mineral weathering	Thermodynamic equilibrium	No	Monthly	No	No	No	No
Rogers[2]	1980		Illuvial	Mineral weathering	Kinetics	Yes		No	No	No	Yes
Zelichenko et al.[45]	1982		Evaporative	Groundwater	Thermodynamic equilibrium	Yes	No	No	No	No	No
McFadden[3]	1982		Illuvial	Precipitation, dust	Kinetics	Yes		No	No	No	No
McFadden et al [4]	1985		Illuvial	Precipitation, dust	Kinetics	Yes	Monthly	No	No	No	No
Marion et al.[5]	1985	CALDEP	Illuvial	Precipitation, dust	Thermodynamic equilibrium	Yes	Daily	No	No	No	No
Mayer[46]	1986	CALSOIL						No	No	No	
Mayer et al. [7]	1988		Illuvial	Dust	Thermodynamic equilibrium	Yes	Monthly	No	No	No	No
Marion et al.[8]	1994	CALGYP	Illuvial	Precipitation, dust	Thermodynamic equilibrium	Yes	Daily	No	No	No	Yes
McFadden et al[9].	1998		Illuvial	Precipitation, groundwater, dust	Kinetics	Yes		No	No	No	No
Hirmas [10]	2010	SLIC	Illuvial	Precipitation, dust	Thermodynamic equilibrium	Yes	Daily	No	Yes	No	No

Revised from Marion and Schlesinger (1994) [8] and Hirmas (2010) [10].

Table S2. Description of the model parameters used in SINOCOM.

Name	Description	Unit	Value	Reference
$logK_H$	K_H was the Henry's constant	mol l^{-1} atm^{-1}	$108.3865+0.01985076T–6919.53/T–40.45154 logT+669365/T^2$	Plummer et al. [47]
$logK_1$	K_1 was the dissociation constant of H_2CO_3	mol l^{-1}	$–356.3094–0.06091964T+21834.37/T +126.8339 logT–1684915/T^2$	Plummer et al. [47]
$logK_2$	K_2 was the dissociation constant of HCO_3^-	mol l^{-1}	$–107.8871–0.03252849T+5151.79/T +38.92561 logT–56371.9/T^2$	Plummer et al. [47]
K_0	Equilibrium constant for the H_2CO_3 and CO_2	-	$1.7\times10^{-4}/K_1$	Wissbrun et al. [48]
$logK_W$	K_W was the dissociation constant of H_2O	mol^2 l^{-2}	$22.801–0.010365T–4787.3/T–7.1321 logT$	Harned et al.[49]
$logK_C$	K_C was the solubility product of $CaCO_3$	mol^2 l^{-2}	$–171.9065–0.077993T+2839.319/T+ 71.595 logT$	Plummer et al. [47]
A	The parameter of ion activity coefficients	-	$0.4883+8.074\times10^{-4}Tc$	Dreybrodt [11]
B	The parameter of ion activity coefficients	-	$0.3241+1.6\times10^{-4}Tc$	Dreybrodt [11]
a_i (Ca^{2+})	The ionic radii of Ca^{2+}	10^{-10} m	5.0	Dreybrodt [11]
a_i (H^+)	The ionic radii of H^+	10^{-10} m	9.0	Dreybrodt [11]
a_i (HCO_3^-)	The ionic radii of HCO_3^-	10^{-10} m	5.4	Dreybrodt [11]
a_i (CO_3^{2-})	The ionic radii of CO_3^{2-}	10^{-10} m	5.4	Dreybrodt [11]
a_i (OH^-)	The ionic radii of OH^-	10^{-10} m	3.5	Dreybrodt [11]
b_i (Ca^{2+})	The parameter of ion activity coefficients	mol^{-1}	0.165	Dreybrodt [11]
fc	Field capacity	cm	Water potential at 33kPa	SoilGrids
wp	Wilting point	cm	Water potential at 1500kPa	SoilGrids
whc	Water holding capacity	cm	fc−wp	SoilGrids
S0	Initial soil moisture conditions	cm	whc/2+wp	SoilGrids

Tc is the temperature (°C), T (K)=Tc+273.16. Revised from Dreybrodt (1998) [11].

Table S3. SIC loss and loss rates under the four climate scenarios from 2015 to 2100.

Depth (cm)	SIC in 2015 (Pg)	Future projection	SIC loss (Tg)	SIC loss of deposit		SIC loss of groundwater		SIC loss rate (%)
				(Pg)	(%)	(Tg)	(%)	
0–10	3.80	SSP1-2.6	307.08	304.70	99.23	2.374	0.77	8.08
		SSP2-4.5	311.64	309.19	99.21	2.450	0.79	8.20
		SSP3-7.0	316.77	314.29	99.22	2.479	0.78	8.34
		SSP5-8.5	321.12	318.58	99.21	2.537	0.79	8.45
0–30	10.98	SSP1-2.6	268.99	266.74	99.16	2.252	0.84	2.45
		SSP2-4.5	273.07	270.76	99.16	2.303	0.84	2.49
		SSP3-7.0	281.58	279.24	99.17	2.341	0.83	2.57
		SSP5-8.5	284.13	281.74	99.16	2.392	0.84	2.59
0–100	30.11	SSP1-2.6	215.54	214.60	99.57	0.917	0.43	0.72
		SSP2-4.5	219.09	218.17	99.58	0.919	0.42	0.73
		SSP3-7.0	229.93	228.98	99.59	0.951	0.41	0.76
		SSP5-8.5	232.15	231.14	97.57	1.007	0.43	0.77
0–200	36.74	SSP1-2.6	208.52	208.49	99.98	0.034	0.02	0.57
		SSP2-4.5	211.17	211.17	100.00	0.018	0.00	0.57
		SSP3-7.0	223.37	223.36	100.00	0.008	0.00	0.61
		SSP5-8.5	225.13	224.98	99.93	0.151	0.07	0.61

Table S4. SIC loss and new SIC formation under the four climate scenarios from 2015 to 2100.

Depth (cm)	SIC in 2015 (Pg)	Future projection	Total SIC loss		Net SIC loss		SIC formation	
			(Tg)	(%)	(Tg)	(%)	(Tg)	(%)
0–10	3.80	SSP1-2.6	317	8.34	307	8.08	10	0.26
		SSP2-4.5	322	8.47	312	8.20	10	0.27
		SSP3-7.0	327	8.62	317	8.34	11	0.28
		SSP5-8.5	333	8.76	321	8.45	11	0.30
0–30	10.98	SSP1-2.6	278	2.53	269	2.45	9	0.08
		SSP2-4.5	282	2.57	273	2.49	9	0.09
		SSP3-7.0	291	2.66	282	2.57	10	0.09
		SSP5-8.5	295	2.69	284	2.59	11	0.10
0–100	30.11	SSP1-2.6	223	0.74	216	0.72	7	0.02
		SSP2-4.5	227	0.75	219	0.73	7	0.02
		SSP3-7.0	238	0.79	230	0.76	8	0.03
		SSP5-8.5	241	0.80	232	0.77	9	0.03
0–200	36.74	SSP1-2.6	215	0.59	209	0.57	7	0.02
		SSP2-4.5	218	0.59	211	0.57	7	0.02
		SSP3-7.0	231	0.63	223	0.61	8	0.02
		SSP5-8.5	234	0.64	225	0.61	9	0.02

Table S5. Predictive model performance of the four machine learning algorithms.

Depth (cm)	Models	R^2
	Random forest	0.90
0–10	Extreme gradient boosting	0.87
	Support vector machines	-3.61
	Random forest	0.89
0–30	Extreme gradient boosting	0.84
	Support vector machines	0.29
	Random forest	0.89
0–100	Extreme gradient boosting	0.87
	Support vector machines	0.26
	Random forest	0.91
0–200	Extreme gradient boosting	0.89
	Support vector machines	0.17

Table S6. Performances of 36 independent prediction models based on the RF model.

Year	Depth (cm)	SIC (0.5°)[1] (Pg)	SIC (1 km)[2] (Pg)	Relative Error (%)	R^2
2020	0–10	3.83	3.77	1.62	0.86
2020	0–30	11.07	10.96	1.04	0.86
2020	0–100	30.65	30.09	1.81	0.85
2020	0–200	37.29	36.72	1.52	0.90
2030	0–10	3.79	3.73	1.67	0.91
2030	0–30	11.04	10.93	1.05	0.87
2030	0–100	30.63	30.07	1.83	0.87
2030	0–200	37.27	36.70	1.53	0.91
2040	0–10	3.76	3.69	1.69	0.89
2040	0–30	11.01	10.90	1.06	0.87
2040	0–100	30.61	30.05	1.83	0.87
2040	0–200	37.25	36.68	1.53	0.90
2050	0–10	3.72	3.66	1.73	0.87
2050	0–30	10.98	10.87	1.07	0.86
2050	0–100	30.59	30.02	1.83	0.85
2050	0–200	37.23	36.66	1.54	0.90
2060	0–10	3.68	3.62	1.75	0.86
2060	0–30	10.95	10.83	1.10	0.87
2060	0–100	30.56	30.00	1.85	0.85
2060	0–200	37.21	36.63	1.55	0.89
2070	0–10	3.65	3.58	1.79	0.85
2070	0–30	10.92	10.80	1.11	0.86
2070	0–100	30.54	29.97	1.85	0.86
2070	0–200	37.18	36.61	1.55	0.90
2080	0–10	3.61	3.55	1.80	0.85
2080	0–30	10.89	10.77	1.11	0.86
2080	0–100	30.51	29.95	1.86	0.87
2080	0–200	37.16	36.58	1.56	0.90
2090	0–10	3.58	3.52	1.86	0.85
2090	0–30	10.86	10.74	1.12	0.87
2090	0–100	30.49	29.92	1.86	0.86
2090	0–200	37.14	36.56	1.56	0.90
2100	0–10	3.55	3.49	1.84	0.85
2100	0–30	10.83	10.70	1.14	0.87
2100	0–100	30.46	29.89	1.87	0.87
2100	0–200	37.11	36.53	1.57	0.89

[1]Chinese SIC stocks of process-based models at a spatial resolution of 0.5°. [2]Chinese SIC stocks derived from a combination of machine-learning and process-based models at a spatial resolution of 1 km.

Table S7. SIC loss and loss rates across climate zones from 2015 to 2100.

Depth (cm)	Climate condition	SIC in 2015 (Pg)	SIC in 2100 (Pg)	SIC loss (Tg)	SIC loss rate (%)
	Humid	0.21	0.10	107.48	51.72
	Dry sub-humid	0.16	0.09	62.65	39.83
0–10	Semi-arid	1.19	1.06	124.32	10.48
	Arid	1.46	1.45	15.85	1.09
	Hyper arid	0.78	0.78	1.34	0.17
	Humid	0.66	0.55	116.38	17.55
	Dry sub-humid	0.51	0.45	59.85	11.84
0–30	Semi-arid	3.90	3.81	88.08	2.26
	Arid	4.21	4.20	8.31	0.20
	Hyper arid	1.70	1.70	0.44	0.03
	Humid	2.06	1.96	100.06	4.85
	Dry sub-humid	1.76	1.71	50.88	2.89
0–100	Semi-arid	12.92	12.86	62.44	0.48
	Arid	10.56	10.56	5.36	0.05
	Hyper arid	2.80	2.80	0.34	0.01
	Humid	2.35	2.25	98.36	4.18
	Dry sub-humid	2.37	2.32	48.92	2.07
0–200	Semi-arid	17.61	17.55	58.30	0.33
	Arid	11.51	11.50	5.26	0.05
	Hyper arid	2.90	2.90	0.34	0.01

Table S8. SIC loss and loss rates by land use from 2015 to 2100.

Depth (cm)	Land use	SIC in 2015 (Pg)	SIC in 2100 (Pg)	SIC loss (Tg)	SIC loss rate (%)
0–10	Forest	0.20	0.12	76.75	38.55
	Cropland	0.61	0.50	104.41	17.21
	Grassland	1.37	1.28	91.58	6.68
	Desert	1.45	1.43	14.24	0.98
0–30	Forest	0.61	0.53	79.64	13.10
	Cropland	1.95	1.87	84.21	4.32
	Grassland	4.35	4.27	76.14	1.75
	Desert	3.49	3.48	11.42	0.33
0–100	Forest	1.95	1.88	68.75	3.53
	Cropland	7.11	7.05	59.48	0.84
	Grassland	12.44	12.38	63.91	0.51
	Desert	6.68	6.67	10.16	0.15
0–200	Forest	2.25	2.18	68.41	3.04
	Cropland	10.85	10.79	54.09	0.50
	Grassland	14.31	14.25	62.83	0.44
	Desert	6.81	6.80	10.10	0.15

Table S9. Quantitative analysis of SIC loss by soil layer across climatic gradients from 2015 to 2100.

Depth (cm)	Humid		Dry sub-humid		Semi-arid		Arid		Hyper arid	
	n[1]	n_SIC loss[2]	n	n_SIC loss	n	n_SIC loss	n	n_SIC loss	n	n_SIC loss
0–10	394	394	249	249	797	797	687	687	256	256
10–20	384	145	247	64	792	50	661	30	195	2
20–30	373	44	244	32	783	48	619	22	157	2
30–40	360	40	243	30	767	43	580	18	126	2
40–50	350	38	237	30	753	40	547	15	93	1
50–60	342	35	233	30	727	38	502	15	79	1
60–70	333	32	225	30	687	38	439	15	58	1
70–80	300	23	196	30	630	37	326	15	50	1
80–90	259	18	151	10	532	33	207	10	31	1
90–100	190	17	119	10	452	29	138	8	16	1
100–110	132	8	101	8	409	29	97	6	10	0
110–120	91	6	92	7	371	28	74	6	10	0
120–130	71	6	76	6	327	28	51	5	7	0
130–140	56	6	64	5	277	28	35	3	3	0
140–150	47	5	59	4	229	18	22	2	2	0
150–160	40	5	50	4	181	18	10	0	1	0
160–170	33	4	42	3	150	18	5	0	0	0
170–180	18	4	38	3	117	12	3	0	0	0
180–190	12	1	31	3	82	9	1	0	0	0
190–200	6	1	26	3	58	9	0	0	0	0

[1]Total number of grids corresponding to the specified climatic conditions. [2]Number of grids showing SIC loss under those climatic conditions.

Table S10. Literature sources of the additional data.

No.	Province	Ca_pre (mol l^{-1})
1	Hainan	3.19×10^{-6}
2	Guangdong	2.40×10^{-5}
3	Fujian	2.96×10^{-5}
4	Chongqing	4.14×10^{-5}
5	Hunan	4.28×10^{-5}
6	Shanghai	4.67×10^{-5}
7	Zhejiang	4.74×10^{-5}
8	Henan	4.83×10^{-5}
9	Tibet	5.21×10^{-5}
10	Anhui	5.58×10^{-5}
11	Guangxi	5.89×10^{-5}
12	Jiangsu	5.90×10^{-5}
13	Yunnan	6.37×10^{-5}
14	Guizhou	6.66×10^{-5}
15	Jilin	6.70×10^{-5}
16	Shandong	6.92×10^{-5}
17	Heilongjiang	7.45×10^{-5}
18	Sichuan	7.90×10^{-5}
19	Beijing	8.31×10^{-5}
20	Liaoning	8.55×10^{-5}
21	Jiangxi	9.47×10^{-5}
22	Hubei	1.19×10^{-4}
23	Qinghai	1.31×10^{-4}
24	Hebei	1.37×10^{-4}
25	Tianjin	1.42×10^{-4}
26	Shanxi	1.85×10^{-4}
27	Shaanxi	1.91×10^{-4}
28	Gansu	2.42×10^{-4}
29	Ningxia	2.42×10^{-4}
30	Xinjiang	3.01×10^{-4}
31	Inner Mongolia	3.04×10^{-4}
32	Taiwan	2.96×10^{-5}
33	Macao	2.96×10^{-5}
34	Hong Kong	2.96×10^{-5}

Table S11. Data sources for the Ca^{2+} data from 2015 to 2025.

1. Zhai Y, Shi Y-L, Ma L-X, Shen L, Yang F, Zhang H-B. Analysis of the chemical characteristics of atmospheric precipitation and the transformation of shallow groundwater during the '13th Five-Year Plan' period. *Modern Chemical Research* 2023; 92. (in Chinese with English abstract)
2. Cheng Y-X, Guan J-W, Fu T et al. Characteristics of atmospheric precipitation chemistry and deposition in typical regions of China during 2000–2021. *China Environmental Science* 2024; 44: 4817–25. (in Chinese with English abstract)
3. Huang H-L. Analysis of precipitation change in Anqing city from 2016 to 2021. *Leather Making Environ Protect Technol* 2022; 23:65. (in Chinese with English abstract)
4. Gao Z, Wang L, Gao L. Chemical characteristics and source apportionment of atmospheric precipitation in Baotou urban area. *Environmental Chemistry* 2024; 43:2490-503. (in Chinese with English abstract)
5. Ye W-X. Characteristics and variation trend of acid precipitation in Fuzhou urban area. *Chemical Engineering & Equipment* 2013; 225. (in Chinese with English abstract)
6. Fei J-Y. Analysis of pollution status and variation trend of atmospheric precipitation in Fushun City. *Science and Technology Forum* 2015; 28. (in Chinese with English abstract)
7. Xiao H-W, Xiao H-Y, Wang Y-L. Chemical characteristics and source apportionment of precipitation in Guiyang. *China Environmental Science* 2010; 30: 1590–6. (in Chinese with English abstract)
8. Ren L, Chen J, Bai Z et al. Ionic composition and source analysis of precipitation at Wuzhi Mountain in Hainan Province and Wuyi Mountain in Fujian Province. *Research of Environmental Sciences* 2012; 25: 404-10. (in Chinese with English abstract)
9. Xie K, Li R, Zhang L et al. Chemical Composition and Source Assessment of Precipitation in Huainan. *Environmental Monitoring in China* 2017; 33: 31–8. (in Chinese with English abstract)
10. Ju, D. G., Ren, D. J., Liu, T. et al. Chemical characteristics and source analysis of precipitation in Huangshi City. *Environmental Chemistry* 2022; 41: 3685–94. (in Chinese with English abstract)
11. Wang S, Sun C, Chen W, et al. Assessing water resource risks in inland river basins of Northwest China from water chemistry and runoff. *Acta Geographica Sinica* 2023; 78: 2763–80. (in Chinese with English abstract)
12. Chen, Q. L., Xu, Y., Yuan, Y. et al. Variation trend and correlation analysis of atmospheric precipitation in Jiangjin District. *Journal of Green Science and Technology* 2022; 24:2 8–31. (in Chinese with English abstract)
13. Zhang, M. Y., Wang, S. J., Zhang, Y. et al. Analysis on the origin and characteristics of chemical composition of precipitation in Jinhua. *Environmental Monitoring in China* 2007; 23: 86–92. (in Chinese with English abstract)

14. Yang Y, Xu H. Investigation on main ion components and their variation characteristics of atmospheric precipitation in Jingmen City. *Low Carbon World* 2017; 7. (in Chinese with English abstract)

15. Liu, D. Y., Yang, R. J., Tian, Q. Ion source analysis of atmospheric precipitation in Kui-Du-Wu area. *Arid Environmental Monitoring* 2017; 31: 126–31. (in Chinese with English abstract)

16. Huang, L. J., Wang, D. X., Wang, J. P. Characteristics and variations analysis on chemical composition of atmospheric precipitation of Kunming. *Environmental Science Survey* 2022; 41: 46. (in Chinese with English abstract)

17. Jia, Y. C., Sun, C. B. Characteristics and causes of precipitation chemical composition in urban area of Lu'an City. *Contemporary Farm Machinery* 2018: 78–80. (in Chinese with English abstract)

18. Li J, Wu HW, Ye H, et al. Variations and source apportionment of precipitation ions in a typical acid deposition region, located in the Poyang Lake watershed: A case study of Mt. Lushan region. *Journal of China Coal Society* 2023; 48: 252–62. (in Chinese with English abstract)

19. Jia, W. X., Li, Z. X. Hydrochemical characteristics and sources of ions in precipitation at the East Qilian Mountains. *Environmental Science* 2016; 37: 3322–32. (in Chinese with English abstract)

20. Xiang H, Liu XL, Zhou YL, et al. Chemical characteristics and source analysis of main components of atmospheric precipitation in Qianjiang District. *Journal of Green Science and Technology* 2020; 16:19–23. (in Chinese with English abstract)

21. Lin, W. H., Zhang, Y. R. Variation trends and sources of the chemical components of atmospheric precipitation in Xiamen during 2021. *Environmental Science and Technology* 2023; 29: 14–21. (in Chinese with English abstract)

22. Ye S, Duan Y, Li Q. The trend of acidity and ion compositions of precipitation during 2000-2019 in Shanghai. *Environmental Chemistry* 2021; 40: 3672–80. (in Chinese with English abstract)

23. Ai, D. S. Chemical composition characteristics and source analysis of atmospheric precipitation in Shanghai. *MSc Thesis*. East China Normal University, 2011. (in Chinese with English abstract)

24. Niu WW, He LY, Hu M. Chemical characteristics of atmospheric precipitation in Shenzhen. *Environmental Science* 2008; 29: 1014-9. (in Chinese with English abstract)

25. Niu, Y. W., He, L. Y., Hu, M. Chemical characteristics of atmospheric precipitation in Shenzhen. *Environmental Science* 2008; 29: 1014–19. (in Chinese with English abstract)

26. Hou, S. Y., Qiu, C. C., Ding, C. et al. Analysis on chemical composition of precipitation and its source apportionment in Xi'an City. *Environmental Chemistry* 2020; 39: 2384–94. (in Chinese with English abstract)

27. Wang WX, Xu PJ. Research progress in precipitation chemistry in China. *Progress in Chemistry* 2009; 21: 266-81. (in Chinese with English abstract)

28. Yang, J., Zhao, Y. Z., Li, J. et al. Advances in research on the characteristics of chemical composition and source analysis of atmospheric precipitation in China. *Environmental Ecology* 2022; 4: 93–100. (in Chinese with English abstract)

Table S12. Data sources for the SIC data from 2015 to 2025.

1. Luo Q, Wang Y, Deng C et al. Distribution of inorganic carbon in soil profile and its relationship with soil saline-alkali property in arid area. *Journal of Soil and Water Conservation* 2017; 31: 240–6. (in Chinese with English abstract)
2. Zhang B-C, Bai Y-F, Li X-B et al. Effects of straw returning farmland, abandoned land and woodland on soil carbon in Hanzhong basin. *Guihaia* 2018; 38: 1081–7. (in Chinese with English abstract)
3. Chen B-M, Feng W-T, Wang Y-G et al. Inorganic carbon sequestration effect of desulfurized gypsum in alkaline soil improvement. *Acta Pedologica Sinica* 2024; 61: 247–57. (in Chinese with English abstract)
4. Xi M, Sui X-M, Kong F-L et al. Distribution and influencing factors of soil inorganic carbon in typical estuarine wetland of Jiaozhou Bay. *Scientia Geographica Sinica* 2018; 38: 1551–9. (in Chinese with English abstract)
5. Chen C, Li J-B. Profile distribution of soil organic and inorganic carbon in urban greenland. *Journal of Agricultural Sciences* 2017; 38: 7–12. (in Chinese with English abstract)
6. Hu D-Y, Zhang H, Su B-W et al. Soil carbon pool allocation dynamics during soil development in the lower Yangtze River alluvial plain. *Environmental Science* 2024; 45: 314–22. (in Chinese with English abstract)
7. Peng K, Zhang F F, Shao Z D et al. Variation and influencing factors of desert-sierozem soil inorganic carbon in different tillage years in the Qitai Oasis, Xinjiang, China. *Journal of Agro-Environment Science* 2024; 43: 91–101. (in Chinese with English abstract)
8. Shang W, Li D-L, Wei L-Y, Ma Q-L, Tang J-N, Li Y-K et al. Effect of Artificial Haloxylon Ammodendron Plantation on Soil Carbon Pools in Arid Desert Region of Shiyang River Basin. *Journal of Soil and Water Conservation* 2018; 32: 191–8. (in Chinese with English abstract)
9. Shu M, Chen D-D, Li Q et al. Spatial stability analysis of surface soil carbon, nitrogen, and phosphorus densities in alpine grasslands in the Sanjiangyuan region. *Acta Agrestia Sinica* 2023; 31: 2748–58. (in Chinese with English abstract)
10. Li L-H, Lei Y-B, Chen Q-J et al. Changes of soil total carbon and plant root characteristics during restoration of Zoige alpine desertified grassland. *Chinese Journal of Ecology* 2023; 42: 282–90. (in Chinese with English abstract)
11. Chen Y-Y, Feng W-T, Kong L et al. Effects of land use on soil inorganic carbon in an inland basin. *Chinese Journal of Ecology* 2019; 38: 3042–9. (in Chinese with English abstract)
12. Wang L-M, Zhang Q, Bai L-H et al. Effects of three artificial vegetation types on soil particle composition and carbon fixation in the Mu Us Sandy Land. *Journal of Soil and Water Conservation Research* 2020; 27: 88–94. (in Chinese with English abstract)
13. Li X-H, Chen W-F, Song X-L et al. Effects of reclamation on distribution of soil carbon and nitrogen in saline soil of the Yellow River Delta. *Acta Pedologica*

Sinica 2018; 55: 1027–35. (in Chinese with English abstract)

14. Sha G-L, Chen Y-X, Wei T-X et al. Distribution of soil carbon and its driving factors under typical restored vegetation in hilly areas of Loess Plateau. *Soils* 2022; 54: 1265–72. (in Chinese with English abstract)
15. Duan D-X, Wang M-Q, Zhao L-Y et al. Wetland Degradation of Tamarix Chinensis Induced Changes in Soil Inorganic Carbon Stocks and Related Environmental Factors in the Yellow River Delta. Chin. *J. Soil Sci.* 2023; 54: 1308–15. (in Chinese with English abstract)
16. Li Q-L, Yan X, Wu X-Z et al. Effects of desertification on soil organic carbons and soil inorganic carbons in desert grassland. *Journal of Soil and Water Conservation* 2019; 33: 98–103. (in Chinese with English abstract)
17. Wang C-Y, Li Y-C, Guan X et al. Compositions and variation rule of soil carbon pool in the coastal area of western Liaoning province. *Geology and Resources* 2021; 30: 173–86. (in Chinese with English abstract)
18. Mao N, Shao M-A, Huang L-M. Distribution characteristics and influencing factors of soil carbon profile along toposequences in Liudaogou watershed. *Journal of Soil and Water Conservation* 2017; 31: 222–230. (in Chinese with English abstract)
19. Zeng T-T, Wang L-M, Yang K et al. Effects of long-term addition of exogenous organic materials on soil inorganic carbon pool. *Chinese Journal of Eco-Agriculture* 2024; 32: 2034–44. (in Chinese with English abstract)
20. Shi LQ, Ma QL, Ma R et al. Soil carbon fractions under typical sand-fixing vegetation on the southern edge of the Tengger Desert. *Arid Zone Research* 2025; 42: 1451-62. (in Chinese with English abstract)
21. Liu Y, Guo W, Liao D et al. Restoration of soil mineral particle-bound inorganic carbon sequestration effect by sand-fixing forest in Mu Us sandy land. *Journal of Soil and Water Conservation* 2025; 39: 31-38, 47. (in Chinese with English abstract)
22. Xi M, Wang Y, Dang C et al. Distribution of soil inorganic carbon and identification of the importance of influencing factors in coastal wetlands of Jiaozhou Bay. *Geographical Science* 2025; 45: 1130-1142. (in Chinese with English abstract)
23. Xu RF, Wang L, Deng L et al. Characteristics of soil carbon, nitrogen, and water of Populus cathayana plantation along different vegetation restoration years in alpine sandy region. *Chinese Journal of Applied Ecology* 2024; 35: 2657–66. (in Chinese with English abstract)
24. Wang X-Y, Fan Q-Y, Zheng Y-Y et al. Characteristics and influencing factors of forest soil carbon stock in an alpine loess region. *Journal of Forest and Environment* 2025; 45: 460–70. (in Chinese with English abstract)
25. Cao S-N, Luo Y-T, Yu B-H, Zhao Y-Z, Wang J-M. Variation and coupling of soil moisture and soil carbon along an elevation gradient in the region of Western Sichuan, China. *Journal of Irrigation and Drainage* 2025; 44:148–56. (in Chinese with English abstract)

26. Shi SW. Effects of long-term application of biochar on soil carbon pool: a case of fluvic cambisol in Shandong Province. *MSc Thesis*. Chinese Academy of Agricultural Sciences, 2019. (in Chinese with English abstract)
27. Yang X. Long-term fertilization effects on soil inorganic carbon changes and mechanism research. *MSc Thesis*. Chinese Academy of Agricultural Sciences; 2020. (in Chinese with English abstract)
28. Su F. Effects of long-term fertilization on soil carbon and nitrogen in different cropping systems of dryland farmland. *Ph.D. Thesis*. Beijing: University of Chinese Academy of Sciences, 2022. (in Chinese with English abstract)
29. Jin X. Characteristics of soil carbon and iron changes under returning farmland to grassland and their contribution to aggregate formation and stability . *MSc Thesis*. Yangling: Northwest A&F University, 2021. (in Chinese with English abstract)
30. Shi X-Q. Distribution characteristics and sources of soil carbon in Shijiazhuang. *MSc Thesis*. Hebei GEO University, 2019. (in Chinese with English abstract)
31. Tong L. Dynamic changes of soil organic carbon and its effect on soil inorganic carbon in erosion-deposition conditions. *Ph.D. Thesis*. Wuhan: Huazhong Agricultural University, 2022. (in Chinese with English abstract)
32. Tian Y-M. Effects of agricultural activities on inorganic carbon in soils of the Henan Yellow Flood Plain. *MSc Thesis*. Zhengzhou University, 2023.
33. Li Q-L. Effects of desertification on soil organic carbon and inorganic carbon in desert grassland. *MSc Thesis*. Ningxia University, 2019. (in Chinese with English abstract)
34. Jiang X-D. Efficiency and mechanism of soil inorganic carbon accumulation following vegetation restoration of shelter forest in Mu Us sandy land. *MSc Thesis*. Northwest A & F University, 2020. (in Chinese with English abstract)
35. Pang X. Responses of plant productivity and soil nutrients of alpine meadows to the disturbance by plateau pika. *Ph.D. Thesis*. Lanzhou University, 2020. (in Chinese with English abstract)
36. Xian Q-L. Study on the change characteristics and coupling relationship of organic carbon and inorganic carbon in soil in the southern slope of Qilian Mountains. *MSc Thesis*. Qinghai Normal University, 2025. (in Chinese with English abstract)

Supplementary References

1. Arkley RJ. Calculation of carbonate and water movement in soils from climatic data. *Soil Science* 1963; **96**: 239–48.

2. Rodgers RJ. A numerical model for simulating pedogenesis in semiarid regions. 1980.

3. McFadden LD. The impacts of temporal and spatial climatic changes on alluvial soils genesis in southern California. 1982.

4. McFadden LD, Tinsley JC. Rate and depth of pedogenic-carbonate accumulation in soils: Formulation and testing of a compartment model. *Geological Society of America Special Paper* 1985; **203**: 23–41.

5. Marion GM, Schlesinger WH, Fonteyn PJ. CALDEP: A regional model for soil $CaCO_3$ (caliche) deposition in southwestern deserts. *Soil Science* 1985; **139**: 468–81.

6. Mayer L. *The Distribution of Calcium Carbonate in Soils: A Computer Simulation Using Program CALSOIL.*, 1986: 86–115.

7. Mayer L, McFadden LD, Harden JW. Distribution of calcium carbonate in desert soils: A model. *Geology* 1988; **16**: 303-306.

8. Marion GM, Schlesinger WH. *Factors of Soil Formation: A Fiftieth Anniversary Retrospective*. Madison, WI, USA: Soil Science Society of America, 1994.

9. McFadden LD, McDonald EV, Wells SG *et al.* The vesicular layer and carbonate collars of desert soils and pavements: formation, age and relation to climate change. *Geomorphology* 1998; **24**: 101–45.

10. Hirmas DR, Amrhein C, Graham RC. Spatial and process-based modeling of soil inorganic carbon storage in an arid piedmont. *Geoderma* 2010; **154**: 486–94.

11. Dreybrodt W. *Processes in Karst Systems*. Berlin, Heidelberg: Springer Berlin Heidelberg, 1988.

12. Liu F, Wu H, Zhao Y *et al.* Mapping high resolution national soil information grids of China. *Science Bulletin* 2022; **67**: 328–40.

13. Sun J, Ye J, Wu W *et al.* Late Oligocene–Miocene mid-latitude aridification and wind patterns in the Asian interior. *Geology* 2010; **38**: 515–8.

14. Yang Y, Galy A, Zhang J *et al.* Dust transport enhanced land surface weatherability

in a cooling world. *Geochem Persp Let* 2023; **26**: 36–9.

15. Raza S, Miao N, Wang P *et al.* Dramatic loss of inorganic carbon by nitrogen-induced soil acidification in Chinese croplands. *Global Change Biology* 2020; **26**: 3738–51.

16. Mi N, Wang S, Liu J *et al.* Soil inorganic carbon storage pattern in China. *Global Change Biology* 2008; **14**: 2380–7.

17. Hartmann J, Moosdorf N. The new global lithological map database GLiM: A representation of rock properties at the Earth surface. *Geochem Geophys Geosyst* 2012; **13**: 2012GC004370.

18. He M, Sakurai K, Wang G *et al.* Physico-chemical characteristics of the soils developed from alluvial deposits on Chongming Island in Shanghai, China. *Soil Science and Plant Nutrition* 2003; **49**: 223–9.

19. Li Y, Fu C, Zeng L *et al.* Carbon accumulation in the red clay layer of the subsoil in a major river delta: Contribution of secondary carbonate. *CATENA* 2020; **186**: 104391.

20. Chen L-M, Zhang G-L, Effland WR. Soil characteristic response times and pedogenic thresholds during the 1000-year evolution of a paddy soil chronosequence. *Soil Science Society of America Journal* 2011; **75**: 1807–20.

21. Dong D, Huang G, Tao W *et al.* Interannual variation of precipitation over the Hengduan Mountains during rainy season. *Intl Journal of Climatology* 2018; **38**: 2112–25.

22. Liu Y, Lu M, Yang H *et al.* Land–atmosphere–ocean coupling associated with the Tibetan Plateau and its climate impacts. *National Science Review* 2020; **7**: 534–52.

23. Raheb A, Heidari A, Mahmoodi S. Organic and inorganic carbon storage in soils along an arid to dry sub-humid climosequence in northwest of Iran. *CATENA* 2017; **153**: 66–74.

24. O'Neill BC, Tebaldi C, Van Vuuren DP *et al.* The scenario model intercomparison project (ScenarioMIP) for CMIP6. *Geosci Model Dev* 2016; **9**: 3461–82.

25. Cheng W, Dan L, Deng X *et al.* Global monthly gridded atmospheric carbon dioxide concentrations under the historical and future scenarios. *Sci Data* 2022; **9**: 83.

26. Yu T, Sun R, Xiao Z *et al.* Estimation of Global Vegetation Productivity from Global

LAnd Surface Satellite Data. *Remote Sensing* 2018; **10**: 327.

27. Gaillardet J, Calmels D, Romero-Mujalli G *et al.* Global climate control on carbonate weathering intensity. *Chemical Geology* 2019; **527**: 118762.

28. Zeng S, Liu Z, Jiang Y *et al.* A greening Earth has reversed the trend of decreasing carbonate weathering under a warming climate. *Nat Commun* 2025; **16**: 2583.

29. Gwiazda RH, Broecker WS. The separate and combined effects of temperature, soil pCO_2, and organic acidity on silicate weathering in the soil environment: Formulation of a model and results. *Global Biogeochemical Cycles* 1994; **8**: 141–55.

30. Zhang G, Wang Q, Zhang F *et al.* Criteria for establishment of soil family and soil series in Chinese Soil Taxonomy (In Chinese). *Acta Pedologica Sinica* 2013; **50**: 826–34.

31. Jacquier DW, Seaton S. Spline tool for estimating soil attributes at standard depths. *CSIRO Land and Water, Australia* 2012.

32. Song X, Yang F, Wu H *et al.* Significant loss of soil inorganic carbon at the continental scale. *National Science Review* 2022; **9**: nwab120.

33. McFadden LD, Amundson RG, Chadwick OA. *Occurrence, Characteristics, and Genesis of Carbonate, Gypsum, and Silica Accumulations in Soils*. Madison, Wis., USA: Soil Science Society of America, 1991.

34. Zeng S, Liu Z, Kaufmann G. Sensitivity of the global carbonate weathering carbon-sink flux to climate and land-use changes. *Nat Commun* 2019; **10**: 5749.

35. Westenbroek SM, Engott JA, Kelson VA *et al. SWB Version 2.0 — A Soil-Water-Balance Code for Estimating Net Infiltration and Other Water-Budget Components*. U.S. Geological Survey, 2018.

36. Kroes JG, van Dam JC. *Reference Manual SWAP Version 3.0.3.* Wageningen, Alterra, 2003.

37. Liu T, Wang L, Feng X *et al.* Comparing soil carbon loss through respiration and leaching under extreme precipitation events in arid and semiarid grasslands. *Biogeosciences* 2018; **15**: 1627–41.

38. Monger HC, Kraimer RA, Khresat S *et al.* Sequestration of inorganic carbon in soil and groundwater. *GEOLOGY* 2015; **43**.

39. Kaufmann G, Dreybrodt W. Calcite dissolution kinetics in the system $CaCO_3$–H_2O–CO_2 at high undersaturation. *Geochimica et Cosmochimica Acta* 2007; **71**: 1398–410.

40. Tao F, Huang Y, Hungate BA *et al.* Microbial carbon use efficiency promotes global soil carbon storage. *Nature* 2023; **618**: 981–5.

41. Morris MD. Factorial sampling plans for preliminary computational experiments. *Technometrics* 1991; **33**: 161–74.

42. Liu X, Sheng H, Jiang S *et al.* Intensification of phosphorus cycling in China since the 1600s. *Proc Natl Acad Sci USA* 2016; **113**: 2609–14.

43. Minasny B, Bandai T, Ghezzehei TA *et al.* Soil Science-Informed Machine Learning. *Geoderma* 2024; **452**: 117094.

44. Zomer RJ, Xu J, Trabucco A. Version 3 of the global aridity index and potential evapotranspiration database. *Sci Data* 2022; **9**: 409.

45. Zelichenko YN, Sokolenko SA. Calculation of the rate of development of new carbonate formations in soils. *Sov Soil Sci* 1982; **14**: 111–7.

46. Mayer L. The distribution of calcium carbonate in soils; a computer simulation using program CALSOIL. *Open-File Report* 1986: 86–115.

47. Plummer LN, Busenberg E. The solubilities of calcite, aragonite and vaterite in CO_2-H_2O solutions between 0 and 90°C, and an evaluation of the aqueous model for the system $CaCO_3$-CO_2-H_2O. *Geochimica et Cosmochimica Acta* 1982; **46**: 1011–40.

48. Wissbrun KF, French DM, Patterson A. The truce ionisation constant of carbonic acid in aqueous solution from 5 to 45°C. *The Journal of Chemical Physics* 1954; **58**: 693–5.

49. Harned HS, Hamer WJ. The ionization constant of water. *Journal of the American Chemical Society* 1933; **55**: 693–5.
